# PTEN and DNA Ploidy Status by Machine Learning in Prostate Cancer

**DOI:** 10.3390/cancers13174291

**Published:** 2021-08-26

**Authors:** Karolina Cyll, Andreas Kleppe, Joakim Kalsnes, Ljiljana Vlatkovic, Manohar Pradhan, Wanja Kildal, Kari Anne R. Tobin, Trine M. Reine, Håkon Wæhre, Bjørn Brennhovd, Hanne A. Askautrud, Erik Skaaheim Haug, Tarjei S. Hveem, Håvard E. Danielsen

**Affiliations:** 1 Institute for Cancer Genetics and Informatics, Oslo University Hospital, NO-0424 Oslo, Norway; karcyl@ous-hf.no (K.C.); andrekle@ifi.uio.no (A.K.); joakim@icgi.no (J.K.); LVLAT@ous-hf.no (L.V.); MANOP@ous-hf.no (M.P.); WKI@ous-hf.no (W.K.); karris@ous-hf.no (K.A.R.T.); trine@icgi.no (T.M.R.); waehre@icgi.no (H.W.); hanne@icgi.no (H.A.A.); erik.haug@siv.no (E.S.H.); tarjei@icgi.no (T.S.H.); 2 Department of Informatics, University of Oslo, NO-0316 Oslo, Norway; 3 Department of Urology, Oslo University Hospital, NO-0424 Oslo, Norway; bjorb@ous-hf.no; 4 Department of Urology, Vestfold Hospital Trust, NO-3103 Tønsberg, Norway; 5 Nuffield Division of Clinical Laboratory Sciences, University of Oxford, Oxford OX3 9DU, UK

**Keywords:** machine learning, prostate cancer, PTEN, DNA ploidy, tumor heterogeneity

## Abstract

**Simple Summary:**

Molecular tissue-based prognostic biomarkers are anticipated to complement the current risk stratification systems in prostate cancer, but their manual assessment is subjective and time-consuming. Objective assessment of such biomarkers by machine learning-based methods could advance their adoption in a clinical workflow. PTEN and DNA ploidy status are well-studied biomarkers, which can provide clinically relevant information in prostate cancer at a low cost. Using a cohort of 253 patients who received radical prostatectomy, we developed a novel, fully-automated PTEN scoring in immunohistochemically-stained tissue slides, which could be used to assess PTEN status in a reliable and reproducible manner. In an independent validation cohort of 259 patients, automatically assessed PTEN status was significantly associated with time to biochemical recurrence after radical prostatectomy, and the combination of PTEN and DNA ploidy status further improved risk stratification. These results demonstrate the utility of machine learning in biomarker assessment.

**Abstract:**

Machine learning (ML) is expected to improve biomarker assessment. Using convolution neural networks, we developed a fully-automated method for assessing PTEN protein status in immunohistochemically-stained slides using a radical prostatectomy (RP) cohort (*n* = 253). It was validated according to a predefined protocol in an independent RP cohort (*n* = 259), alone and by measuring its prognostic value in combination with DNA ploidy status determined by ML-based image cytometry. In the primary analysis, automatically assessed dichotomized PTEN status was associated with time to biochemical recurrence (TTBCR) (hazard ratio (HR) = 3.32, 95% CI 2.05 to 5.38). Patients with both non-diploid tumors and PTEN-low had an HR of 4.63 (95% CI 2.50 to 8.57), while patients with one of these characteristics had an HR of 1.94 (95% CI 1.15 to 3.30), compared to patients with diploid tumors and PTEN-high, in univariable analysis of TTBCR in the validation cohort. Automatic PTEN scoring was strongly predictive of the PTEN status assessed by human experts (area under the curve 0.987 (95% CI 0.968 to 0.994)). This suggests that PTEN status can be accurately assessed using ML, and that the combined marker of automatically assessed PTEN and DNA ploidy status may provide an objective supplement to the existing risk stratification factors in prostate cancer.

## 1. Introduction

Machine learning, and in particular deep learning, is expected to transform many areas of medicine due to its unmatched capability to make accurate and objective predictions [1]. These methods have proven particularly useful in medical image analysis and have great potential to improve the assessment of diagnostic and prognostic biomarkers in terms of efficiency and reproducibility [2]. Convolution neural networks (CNNs) are a fundamental class of deep learning networks that can be trained to detect, segment, and classify objects using large learning data sets [1,3]. CNNs are well-suited to perform complex visual recognition tasks, such as tumor detection, Gleason grading [4,5], scoring of tissue stains [6,7], as well as determining prognosis [8], and are emerging as a core method in medical image analysis.

Localized prostate cancer (PCa) is a heterogeneous disease with a highly variable clinical outcome [9]. Although several useful prognostic tools combining clinicopathological parameters are available, additional objective biomarkers are needed to further improve risk stratification [10]. Currently, no molecular tissue-based PCa biomarker is recommended for routine clinical use [11].

Chromosomal instability (CIN)—a high rate of loss or gain of whole or parts of chromosomes—is a form of genomic instability observed in most human cancers. It is associated with intratumor heterogeneity and a more aggressive cancer phenotype [12,13]. CIN status can be inferred from measurements of DNA ploidy (cellular DNA content), which is a prognostic biomarker in PCa (reviewed in [14]). DNA ploidy status is best assessed using DNA image cytometry, where the correct and reproducible subclassification of nuclei can be provided by a machine learning-based method. However, the resolution of DNA image cytometry is insufficient to detect additions or deletions of small chromosomal fragments. Loss of the phosphatase and tensin homolog (PTEN) tumor suppressor gene is one of the most common genomic alteration in PCa, and it has been consistently reported to be associated with adverse clinical outcomes (reviewed in [15]). Lennartz et al. demonstrated that combining assessment of DNA ploidy by flow cytometry and deletions of PTEN and 6q15 by fluorescence in situ hybridization (FISH) provided an independent prognostic biomarker in a large cohort of PCa patients. Since PTEN protein loss is highly concordant with gene deletion, PTEN status can be readily obtained by immunohistochemistry (IHC), which is more feasible to adapt to the pathology workflow compared to FISH [16]. However, manual scoring of IHC-stained slides is very time consuming and subjective, and the published computer-aided PTEN scoring methods [17,18] are inadequate to mitigate these issues. 

The aim of this study was to develop a fully automated method for PTEN scoring of IHC-stained slides using CNNs and to determine its prognostic value in patients treated with radical prostatectomy (RP). The method was trained and internally tested using a discovery cohort and validated in an independent cohort according to a predefined protocol that precisely described the primary analysis. As a secondary analysis, we investigated whether combining the automatic PTEN assessment with automatically assessed DNA ploidy status would improve prognostication. 

## 2. Materials and Methods 

### 2.1. Patients

The discovery and validation cohorts were both comprised of patients with PCa who underwent RP at the Norwegian Radium Hospital, Oslo, a tertiary comprehensive cancer center in Norway. The patients in the two cohorts were operated on by different surgeons at largely disjointed time periods (46 out of the 512 (9%) patients were operated on during the overlapping time period) and in general using different surgical techniques. According to the convention in the medical statistics community, such an approach represents a type of external validation called narrow validation, which may be considered intermediate between broad and internal validation [19]. Each prostate gland was processed into a series of 3–5 mm thick formalin-fixed, paraffin-embedded tissue blocks. Both cohorts are described in detail in the study protocol (File S1 page 1–5). The study was approved by the Norwegian Regional Committees for Medical Research Ethics South-East region (REK numbers S-07443a and 2013/476). Gleason scores (GSs) of the tumors were assessed in the clinical routine for all patients in the validation cohort. All study specimens were centrally reviewed, at different time points, by an experienced uropathologist (LV) using the updated 2005 International Society of Urological Pathology (ISUP) guidelines [20,21] in the discovery cohort and the 2014 ISUP guidelines [22] in the validation cohort. The definitions of Gleason grade patterns in the updated 2005 and 2014 ISUP consensus are similar. The only difference is the recommendations on grading of glomeruloid glands, which are an extremely rare feature in prostate tumors [23]. Gleason scores were classified into Gleason grade groups (GGGs) [22].

### 2.2. Discovery Cohort and Test Subset

Of the 317 patients operated on with open retropubic prostatectomy between 1987 and 2005 by one surgeon (HW), 10 were excluded due to preoperative therapy (*n* = 1), death from postoperative complications (*n* = 1), loss to follow-up (*n* = 1), or no tumor material available (*n* = 7). 

A subset of 185 blocks from 93 non-excluded patients was used to develop a CNN to detect the tumor region in which the PTEN score was assessed. This subset was randomly split on the patient level into a train subset containing 70% of the patients and a tune subset containing the other 30%. The train subset contained 129 blocks from 65 patients and was used to train the tumor detector. The tune subset contained 56 blocks from 28 patients and was used to select model hyperparameters, in particular to determine when to cease training.

Another CNN was developed to detect and segment tumor cells and classify them as PTEN-positive or PTEN-negative. This development used a subset of 34 blocks from 34 patients, which were randomly split into a train and a tune subset, again targeting a 70:30 split. The resulting train subset contained 24 blocks, and the tune subset contained the remaining 10 blocks.

A test subset of 253 non-excluded patients with three available tumor-containing blocks was used to evaluate the performance of the automatic PTEN scoring method (protocol page 30–34). None of the 34 patients used for developing the PTEN classifier were included in the test subset, whereas 50 patients were included in both the dataset used for developing the tumor detector (i.e., the 93 patients) and the test subset. Different thresholds for dichotomizing the automatic PTEN scores were evaluated in the test subset (i.e., the 253 patients), and the decision to use 50% as the threshold in the validation was based on these results (protocol page 33–34).

### 2.3. Validation Cohort

Of the 287 patients operated on with open retropubic prostatectomy (*n* = 75) or robot-assisted prostatectomy (*n* = 182) between 2001 and 2006 by one surgeon (BB), 28 were excluded due to missing patient consent (*n* = 21), missing or less than six weeks of follow-up (*n* = 4), and no tumor material available (*n* = 3). Three tumor-containing blocks were analyzed for each of the 259 eligible patients. 

### 2.4. Immunohistochemistry, Scanning of Tissue Slides, and Manual PTEN Scoring 

Monoclonal PTEN antibody (1:400, 138G6, Cell Signaling Technology, Danvers, MA, USA) was applied on 3 μm tissue sections after heat-induced epitope retrieval, as previously described [24]. IHC-stained slides were scanned on a NanoZoomer XR slide scanner (Hamamatsu Photonics, Hamamatsu, Japan) at the highest resolution available (termed 40x). The resulting whole-slide images (WSIs) typically contained an order of 100.000 × 100.000 pixels, each representing a physical size of 0.227 × 0.227 µm. All the WSIs were quality controlled, and slides were rescanned if they were out of focus. PTEN expression was manually scored at 10% intervals by two observes (Karolina Cyll (KC) and Elin Ersvær), blinded to clinicopathological data. Cells were considered PTEN-negative if the cytoplasmic and nuclear staining was absent or decreased compared with internal positive controls (benign glands and/or stroma), as previously described [16,25]. PTEN expression was not scored when the intensity of the staining was weak or absent in the internal positive controls or when ≥95% of the tumor area had fallen off during sample preparation. The correlation between the manual scores obtained by the two observers was strong (Pearson’s r = 0.916, 95% CI 0.903 to 0.927). Survival analysis of each observer’s PTEN scores is presented in Figure S1. A consensus score was used in further analyses.

### 2.5. DNA Image Cytometry 

Preparation of nuclear monolayers was performed according to a modified Hedley method [26]. Identification of representative epithelial and stromal (reference) nuclei and DNA ploidy histogram classification into diploid, tetraploid, or aneuploid was done automatically using PWS Classifier software (Room4 Ltd., Sussex, UK). The software makes use of support vector machines, a machine learning technique, trained with manual cell classifications as references to discard non-intact nuclei (i.e., cut, folded, and connected) and to classify cell types based on morphological features and pixel-based image metrics extracted from the cell images [27,28] (see File S1 page 9–10 for details). 

### 2.6. Automatic PTEN Scoring

**Figure 1:**
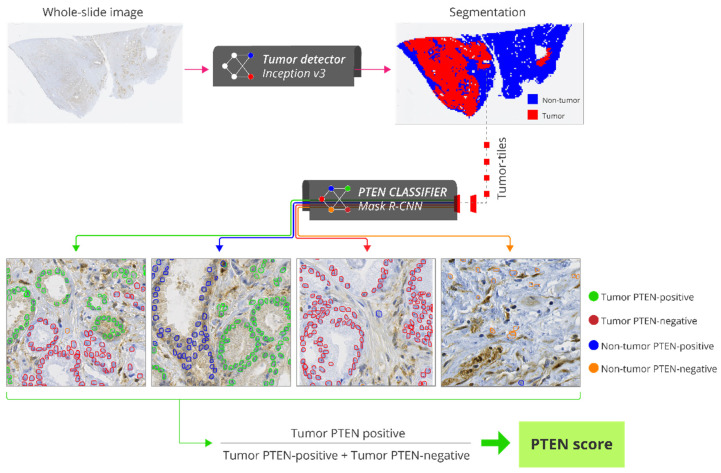
Pipeline for fully-automatic PTEN scoring. Each whole slide image (WSI) of an IHC-stained slide is partitioned into smaller, non-overlapping regions called tiles with 800 × 800 pixels (40x lens). The tiles are classified as tumor or non-tumor by the tumor detector. Tumor tiles are processed by the PTEN classifier which detects and quantifies tumor PTEN-positive, non-tumor PTEN-positive, tumor PTEN-negative and non-tumor PTEN-positive cells. PTEN score for a WSI is calculated as the ratio between the number of tumor PTEN-positive cells and the total number of tumor PTEN-positive and PTEN-negative cells. Abbreviations: PTEN = phosphatase and tensin homologue.

The automatic scoring method consisted of three steps. First, each WSI was partitioned into smaller, non-overlapping regions, called tiles, measuring 800 × 800 pixels. Next, each tile was classified as tumor or non-tumor by the tumor detector. Finally, the PTEN classifier detected and segmented tumor cells in the remaining tumor tiles and classified them as PTEN-positive or PTEN-negative. The entire system thus provided a count of PTEN-positive and PTEN-negative tumor cells without any human interaction (Figure 1). The PTEN score for a WSI was calculated as the ratio between the number of positive cells and the total number of positive and negative cells. The score for a patient was calculated as the average score of all its WSIs. 

The training and tuning of the tumor detector and PTEN classifier are described in detail in the File S1 (page 12–29). Briefly, the tumor detector was developed using the Inception v3 classification CNN [29]. The train subset of 129 WSIs from 65 patients contained 881,418 tiles, whereas the tune subset of 56 WSIs from 28 patients contained 332,211 tiles. A tile was classified as a tumor tile if its center position was inside the manual tumor annotations performed in the WSI; otherwise, it was classified as a non-tumor tile. This resulted in 241,170 (27%) tumor tiles and 640,248 (73%) non-tumor tiles in the train subset, and 97,587 (29%) tumor tiles and 234,624 (71%) non-tumor tiles in the tune subset. In order to represent cases with technical failures, 10 of the 185 WSIs were IHC-stained with lower PTEN antibody concentration (1:1200), and another 10 were IHC-stained without PTEN antibody. Tumor areas in these 20 WSIs were not annotated in order to allow the network to learn to exclude them. The proportion of tiles correctly classified as tumor/non-tumor was 0.957 in the train subset and 0.938 in the tune subset. 

The PTEN classifier was developed using the Mask R-CNN instance segmentation network [30]. The train subset of 24 WSIs from 24 patients consisted of 2160 tiles, and the tune subset of 10 WSIs from 10 patients consisted of 900 tiles. Contours of 77,777 tumor nuclei from the 3060 tiles were manually drawn to learn the network to identify cells. Each cell was labeled as PTEN-positive (cytoplasmic and/or nuclear staining present) or PTEN-negative (cytoplasmic and nuclear staining absent) by a trained cell biologist (KC). The train subset consisted of 46,434 (81%) PTEN-positive and 11,146 (19%) PTEN-negative cells, whereas the tune subset consisted of 17,396 (86%) PTEN-positive and 2801 (14%) PTEN-negative cells. 

The development of the PTEN classifier was an iterative process. First, a model was trained and tuned using the 3060 manually annotated tiles from the 34 WSIs, resulting in a mean average precision [31] of 0.856 in the train subset and 0.687 in the tune subset (File S1 page 19–20). To improve the model’s ability to discriminate tumor and non-tumor cells, we applied the initial model to the 3060 tiles, and the detections that did not overlap with the manual annotations were reclassified (KC) into four classes: tumor PTEN-positive, tumor PTEN-negative, non-tumor PTEN-positive, or non-tumor PTEN-negative. This refinement of the annotations in the tiles from the 34 WSIs allowed for the inclusion of tumor cells that were missed during the initial manual annotation. In addition, this allowed for the inclusion of non-tumor PTEN-positive and PTEN-negative cells that were not annotated manually but rather incorrectly identified as tumor cells by the first model to improve the network’s ability to differentiate between tumor and non-tumor cells. The tiles from the 34 WSIs were coupled with the updated annotations, including the four classes and a background class, and used to train a second (and final) model. The mean average precision of this final model was 0.835 in the train subset and 0.705 in the tune subset (File S1 page 22–23).

### 2.7. Statistical Analyses

The study was performed in compliance with the Reporting Recommendations for Tumor Marker Prognostic Studies (REMARK) [32]. A study protocol describing the independent validation was predefined in accordance with the Protocol Items for External Cohort Evaluation of a deep learning System (PIECES) [33]. The primary and secondary analyses were planned prior to the evaluations of the independent validation cohort and are described in the protocol (File S1 page 35–37). The primary analysis was the assessment of the prognostic value of the automatically assessed dichotomous biomarker of PTEN status in the validation cohort by computing its hazard ratio (HR) with a 95% confidence interval (CI) in univariable Cox proportional hazard regression analysis and the *p*-value of the Mantel–Cox log-rank test. The endpoint was biochemical recurrence (BCR), defined as a single PSA ≥ 0.4 ng/mL. Time to BCR (TTBCR) was calculated from primary surgery to BCR or to the date of the final PSA registration (24 June 2020). In the analysis of the test subset of the discovery cohort, the endpoint was time to recurrence defined in accordance with Punt et al. [34], calculated from primary surgery to recurrence or to non-related death or the last date of follow-up (31 December 2008). Survival curves were depicted with the Kaplan–Meier method and compared using the Mantel–Cox log-rank test. The marker of interest and established prognostic markers were included in the multivariable model and evaluated using the Wald χ^2^ test with the Cox proportional hazards model. The CI of the area under the receiver operating characteristic curve (AUC) and Harrell’s concordance index (c-index) were computed as the bias-corrected and accelerated (BCa) percentile interval over 10,000 bootstraps. PTEN and DNA ploidy status were integrated with the Cancer of the Prostate Risk Assessment Post-Surgical (CAPRA-S) score by adding 1 point if PTEN-low and 1 point if non-diploid. In order to test the difference in c-index between the standard and the updated CAPRA-S score, a two-sided *p*-value was calculated as 1 minus the confidence level of the largest BCa CI that did not contain 0. Correlations between the automatic and the manual PTEN scores were evaluated using Pearson’s correlation coefficient. The AUC was used to measure the performance of the automatic PTEN scoring method, using manual PTEN scores dichotomized using the 50% threshold as the ground truth. Fisher’s exact test, Kruskal–Wallis H, and Mann–Whitney *U* tests were used to evaluate associations. Two-sided *p*-values <0.05 were considered statistically significant. Statistical calculations were performed using Stata/MP 16.1 (StataCorp, College Station, TX, USA).

## 3. Results

### 3.1. Test Subset

Clinicopathological characteristics of patients included in the test subset are summarized in Table S1. Approximately half of the patients (46%) were in the CAPRA-S low- or intermediate-risk group. Patients with PTEN-low (<50%) had a significantly shorter time to recurrence compared to patients with PTEN-high (≥50%) in univariable analysis (HR = 1.96, 95% CI 1.27 to 3.02, *p* < 0.001 and c-index = 0.578, 95% CI 0.527 to 0.634).

### 3.2. Validation Cohort

The patients in the validation cohort had a median age of 62 (interquartile range (IQR) 59–66) years, and the majority (77%) were in the CAPRA-S low- or intermediate-risk group (Table 1). BCR occurred in 71 patients after a median of 2.4 (IQR 0.3–5.3) years. The median follow-up time for patients that did not experience BCR was 9.2 (IQR 7.9–11.1) years.

**Figure 2:**
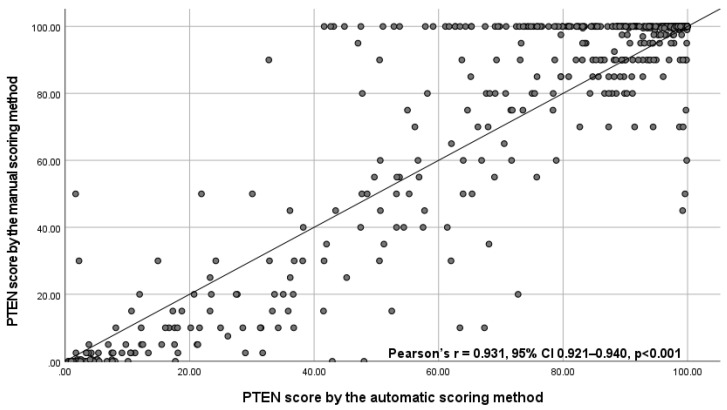
Scatterplot with correlation coefficient between the automatic PTEN score and the manual PTEN scores for 694 whole slide images with valid scores obtained by both scoring methods. Abbreviations: CI = confidence interval, PTEN = phosphatase and tensin homologue, SD = standard deviation.

Of the 777 IHC-stained slides from 259 patients, automatic PTEN scores were obtained for 735 WSIs from 259 patients and manual PTEN scores for 704 WSIs from 256 patients. Fewer WSIs were scored manually due exclusion of cases with technical failures following quality control of the staining, which was not performed for automatic scoring (Figure S2). The correlation between the automatic and manual scores of the same WSIs was strong (Pearson’s r = 0.931, 95% CI 0.921 to 0.940) (Figure 2). The AUC was 0.987 (95% CI 0.968 to 0.994). 

The continuous PTEN scores for patients were associated with TTBCR, with an estimated HR for a 10 percentage point decrease in the PTEN fraction of 1.21 (95% CI 1.12 to 1.29, *p* < 0.001) for the automatic PTEN scores and 1.19 (95% CI 1.12 to 1.27, *p* < 0.001) for the manual PTEN scores. These associations remained statistically significant in multivariable analyses (HR = 1.08, 95% CI 1.00 to 1.16, *p* = 0.045 and HR = 1.09, 95% CI 1.01 to 1.19, *p* = 0.035, respectively).

**Figure 3:**
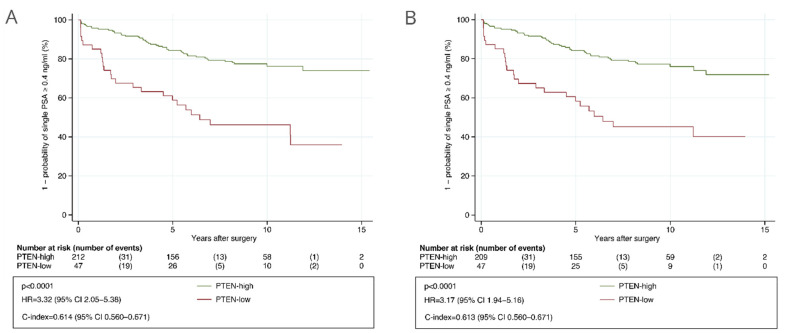
Kaplan–Meier analysis of time to biochemical recurrence after radical prostatectomy stratified by PTEN status in the validation cohort. (**A**) Determined with automatic PTEN scoring. (**B**) Determined with manual PTEN scoring after consensus. Abbreviations: CI = confidence interval; HR = hazard ratio; PTEN = phosphatase and tensin homolog.

In the primary analysis, patients automatically assessed as PTEN-low had a significantly higher risk of BCR compared to patients automatically assessed as PTEN-high (HR = 3.32, 95% CI 2.05 to 5.38 and c-index = 0.614, 95% CI 0.560 to 0.671, Figure 3A). A similar prognostic value was observed for the manually assessed PTEN status (HR = 3.17, 95% CI 1.94 to 5.16, Figure 3B). Automatically assessed PTEN status was associated with TTBCR in CAPRA-S high-risk patients and in those with GGG ≤ 3 tumors in the analyses including routine GSs (Figure S3), and additionally in CAPRA-S low-risk patients and in those with GGG > 3 tumors in the analyses including centrally reviewed GSs (Figure S4). It was also significantly associated with TTBCR in multivariable analysis including routine GSs (HR = 2.24, 95% CI 1.24 to 4.07, *p* = 0.008) and centrally reviewed GSs (HR = 1.86, 95% CI 1.01 to 3.40, *p* = 0.045). The c-index for the standard CAPRA-S score including routine GSs was 0.807 (95% CI 0.750 to 0.851) and 0.812 (95% CI 0.755 to 0.855) for the CAPRA-S score integrated with PTEN status. The difference between these c-indices was 0.005 (95% CI −0.003 to 0.016).

Non-diploid tumors were observed in 70 (27%) of the 259 patients. Patients with non-diploid tumors had a significantly shorter TTCBR compared to those with diploid tumors in univariable analysis (HR = 1.98, 95% CI 1.23 to 3.19, *p* = 0.004 and c-index = 0.581, 95% CI 0.525 to 0.641) but not in multivariable analysis including routine GSs (HR = 1.26, 95% CI 0.75 to 2.11, *p* = 0.37) or centrally reviewed GSs (HR = 1.23, 95% CI 0.73 to 2.11, *p* = 0.43).

**Figure 4:**
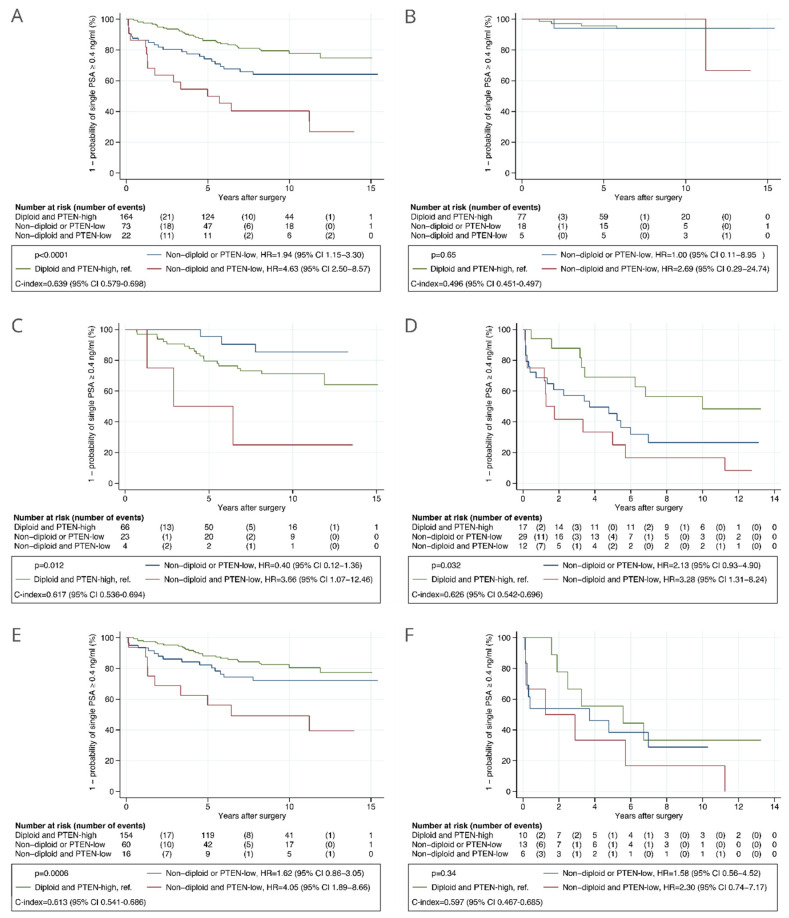
Kaplan–Meier analysis of time to biochemical recurrence after radical prostatectomy stratified by the combined automatically assessed PTEN and DNA ploidy status in the validation cohort. (**A**) All patients. (**B**) Patients with low risk as given by CAPRA-S score. (**C**) Patients with intermediate risk as given by CAPRA-S score. (**D**) Patients with high risk as given by CAPRA-S score. (**E**) Patients with GGG ≤ 3 tumors. (**F**) Patients with GGG > 3 tumors. Routine Gleason scores were used in the analyses. Abbreviations: CAPRA-S = Cancer of the Prostate Risk Assessment Post-Surgical score; C-index = concordance index; CI = confidence interval; GGG = Gleason grade group; HR = hazard ratio; PTEN = phosphatase and tensin homolog.

Combining the PTEN and DNA ploidy status, a high-risk of BCR was observed for patients with both PTEN-low and non-diploid tumors (HR = 4.63, 95% CI 2.50 to 8.57) and intermediate-risk for patients with either PTEN-low or non-diploid tumors (HR = 1.94, 95% CI 1.15 to 3.30), when compared to patients that had PTEN-high and diploid tumors (Figure 4A). The c-index of the combined marker was 0.639 (95% CI 0.579 to 0.698). The combined marker was associated with TTBCR in CAPRA-S intermediate- and high-risk patients and in those with GGG ≤ 3 tumors in the analyses including routine GSs (Figure 4), and only in CAPRA-S high-risk patients in the analyses including centrally reviewed GSs (Figure S5). The association of the combined marker with TTBCR was statistically significant in multivariable analysis including routine GSs (HR = 2.82, 95% CI 1.34 to 5.94 in high-risk and HR = 1.11, 95% CI 0.62 to 1.99 in intermediate-risk when compared to low-risk, *p* = 0.017) (Table 2) and not significant in multivariable analysis including centrally reviewed GSs (HR = 2.22, 95% CI 1.04 to 4.74 in high-risk and HR = 1.12, 95% CI 0.61 to 2.04 in intermediate-risk when compared to low-risk, *p* = 0.10) (Table S2A). The combined marker was associated with TTBCR after adjusting for the CAPRA-S risk groups computed using routine GSs (*p* = 0.011, Table 2) and centrally reviewed GSs (*p* = 0.017, Table S2B). The difference in c-index between the standard CAPRA-S score including routine GSs and the CAPRA-S score integrated with the combined marker was −0.004 (95% CI −0.016 to 0.009).

## 4. Discussion

To our knowledge, this is the first study reporting the development of a fully automated method for scoring PTEN using IHC-stained slides, and the first study of the prognostic value of PTEN status in PCa that mitigates the challenges posed by intratumor heterogeneity. The method correlated strongly with manual scoring and was applied in three tumor-containing blocks for each patient. Using a reliable validation setup with predefined analyses, we have shown that patients with automatically assessed PTEN-low had a three-fold increased risk of BCR after RP compared to those with PTEN-high. This association remained statistically significant in multivariable analysis with established prognostic markers. Furthermore, we observed improved risk stratification when PTEN status was combined with DNA ploidy status assessed with another machine learning-based method.

We have shown that machine learning-based methods can automatically detect and quantify individual PTEN-positive and PTEN-negative tumor cells, providing a robust and accurate assessment of PTEN score. While it is difficult to explain how many modern machine learning approaches obtain their predictions [35,36], the proposed approach for assessment of PTEN score is inherently more easily explained, as it is directly analogous to manual scoring. The basis of the automatic PTEN scores can be easily verified since the method provides a visual presentation of the tumor tiles as well as a localization and classification of the detected cells. Our approach could be used to develop scoring methods for scoring of other biomarkers in IHC-stained slides.

A recent study presented a method using CNNs to detect areas with PTEN-negative cells in tissue microarray (TMA) slides [17]. However, the method could not be used to predict PTEN scores, as it did not detect areas with PTEN-positive cells nor the individual PTEN-negative cells. The method required fine-tuning to improve the correlation with manual annotations in the external TMA validation cohort, even though these slides were IHC-stained using the same conditions as the training cohort. In general, a challenge using TMAs is that they are not directly comparable to RP or biopsy specimens where the proportion of tumor to non-tumor tissue is more variable. As our method was developed in WSIs from RP specimens, which represent tumors better than TMAs, we consider it to be more feasible to implement in the clinical setting. 

Unlike the published computer-aided PTEN scoring methods [17,18], our method is fully automated; hence, it does not require any input from skilled personnel to manually annotate tumor areas or to ensure the quality of IHC-stained slides. Such requirements do not only entail time-consuming manual labor but can also introduce substantial inter- and intra-observer variation. Tumor areas in PCa WSIs need to be carefully annotated to exclude non-tumor cells, which are often intermixed with the tumor cells and may confound the PTEN score. Our method includes multiple steps to ensure that only tumor cells are used to calculate the PTEN score, both by excluding non-tumor regions as well as benign epithelial or stromal cells within the tumor regions. 

Areas in which IHC staining appeared to be absent in tumor cells and weak in internal controls were the main source of discrepancies between the manual and automatic PTEN scores. Such areas were considered to represent technical failures and therefore omitted when scoring manually, whereas some were scored by the automatic method, resulting in lower automatic PTEN scores for some WSIs (Figure 2). Our method could perhaps be further improved by using a larger set of WSIs representing true technical failures, where the presence of PTEN protein had been confirmed by other assays. However, the interpretation of staining intensity is subjective, and some of these tumor areas might have been wrongly considered as technical failures when scoring manually. Overall, the manual and automatic PTEN scores were strongly correlated and provided similar prognostic information when analyzed as continuous as well as dichotomous markers. 

As far as we know, all previous studies on the prognostic value of PTEN status in RP specimens were performed using TMAs [16,18,25,37] or a single tumor-containing block per patient [38]. As PTEN protein expression displays considerable intratumor heterogeneity [24], such sparse sampling may lead to a misclassification of PTEN status. To better represent intratumor heterogeneity in prostate tumors, we assessed PTEN status in WSIs from three different tumor-containing tissue blocks for each patient.

There is currently no consensus on how to dichotomize PTEN scores in IHC studies, and thresholds of 90% [37,38], 50% [39], and 10% [16,25] PTEN-negative cells have previously been used for manual PTEN scores. The study by Jamaspishvili et al. [18] assessed PTEN scores semi-automatically and defined the threshold in a discovery cohort and validated it in an independent validation cohort. This study was performed using TMAs, and the threshold of 65% PTEN-negative cells was selected using maximally log-rank statistics, and BCR defined as two PSA values ≥ 0.2 ng/mL as an endpoint. We selected the 50% threshold to dichotomize PTEN status because this threshold provided relatively large HRs and c-indices across several clinically relevant endpoints in the test subset and was considered to be suited for contemporary cohorts where fewer patients have advanced disease at the time of surgery compared to those in the test subset (File S1 page 33–34). 

The automatic PTEN scoring method was validated in the independent cohort using BCR as the endpoint, which is a limitation of our study. BCR is an intermediate endpoint, which does not always translate into clinical recurrence or PCa death [40]. However, we defined BCR as a single PSA ≥ 0.4 ng/mL, which is suggested to exclude most patients with detectable PSA who are unlikely to progress [41,42,43]. We observed larger HR and c-indices of PTEN status in the validation cohort than in the test subset. This could be due to the use of BCR as an endpoint and the fact that tumors in the test subset were more advanced compared to those in the validation cohort, and PTEN status is suggested to provide stronger prognostic information in patients with less advanced tumors [15,18]. 

The combination of automatically assessed PTEN and DNA ploidy status provided stronger prognostic information than either marker alone when comparing the HRs and c-indices. Patients with both PTEN-low and non-diploid tumors had a 4.63 times increased risk of BCR compared to those with PTEN-high and diploid tumors, suggesting that these two alterations together may result in a more aggressive tumor phenotype. However, the addition of the combined marker to CAPRA-S score did not provide a significant increase in prognostic discrimination in terms of the c-index. The CAPRA-S score includes GS and factors used to determine tumor stage, which are strong prognostic parameters in the postoperative setting [22,44], but their assessment is subjective and is best when performed by experts [45]. Importantly, our cohorts comprised patients operated on at the tertiary comprehensive cancer center, where the routine pathological examination of RP specimens is likely better than in the community hospitals [46], and central review of GSs was performed by a highly experienced uropathologist. 

Automatic measurements of PTEN and DNA ploidy status may be particularly useful in the preoperative setting, where the complete GS and tumor staging information is not available. Therefore, prediction of patient outcomes by pathological assessment is less accurate in the preoperative setting compared to the postoperative setting [47,48,49]. Assessment of cribriform morphology and/or intraductal carcinoma on diagnostic biopsies is suggested to refine the current GGGs and aid in selection of patients for active surveillance [50]. These morphological characteristics were shown to be associated with increased genomic instability and PTEN loss [51,52], but still their assessment suffers from inter-observer variation [53,54]. A limitation of our PTEN scoring method is that it was developed in RP specimens and, thus, it may not be directly applicable for use on biopsies, where tumor areas are smaller. On the other hand, RP specimens provide a large amount of data, which is beneficial when training CNNs and which would be challenging to obtain from biopsies. However, we hypothesize that our PTEN scoring method could be optimized for use in a biopsy setting by applying transfer learning [55,56] and a small discovery dataset of biopsy samples. 

## 5. Conclusions

In conclusion, we have developed a fully automated method for robust and accurate assessment of PTEN score in IHC-stained slides, which could replace manual scoring by human experts. Patients with PTEN-low had significantly shorter TTBCR compared to those with PTEN-high. The combination of PTEN and DNA ploidy status, both assessed using machine learning-based methods, further improved risk stratification.

## Figures and Tables

**Table 1:** Clinicopathological characteristics for all patients in the validation cohort stratified by the combined DNA ploidy and PTEN status.

Characteristic	All	PTEN-High and Diploid	PTEN-Low or Non-Diploid	PTEN-Low and Non-Diploid	*p* Value *
Patients	259	164 (63%)	73 (28%)	22 (8%)	
Age at surgery, years	62 (59–66)	61 (58–65)	65 (60–69)	64 (60–66)	0.002
Preoperative PSA, ng/mL	8.3 (6.5–11.4)	8.0 (6.4–11.0)	9.0 (6.9–12.7)	7.8 (6.4–10.0)	0.18
Missing	1 (0%)	0	1 (1%)	0	
Preoperative PSA					0.44
≤6 ng/mL	54 (21%)	37 (23%)	12 (17%)	5 (23%)	
>6 ng/mL and ≤10 ng/mL	123 (47%)	78 (48%)	32 (44%)	13 (59%)	
>10 ng/mL and ≤20 ng/mL	73 (28%)	45 (27%)	25 (35%)	3 (14%)	
>20 ng/mL	8 (3%)	4 (2%)	3 (4%)	1 (5%)	
Missing	1 (0%)	0	1 (0%)	0	
Gleason grade group ^†^					<0.001
1 (GS 6)	3 (1%)	1 (1%)	2 (3%)	0	
2 (GS 3 + 4)	153 (59%)	117 (71%)	31 (42%)	5 (23%)	
3 (GS 4 + 3)	54 (21%)	31 (19%)	19 (26%)	4 (18%)	
4 (GS 8)	12 (5%)	5 (3%)	4 (6%)	3 (14%)	
5 (GS 9–10)	37 (14%)	10 (6%)	17 (23%)	10 (45%)	
Gleason grade group ^‡^					<0.001
1 (GS 6)	77 (30%)	64 (39%)	12 (16%)	1 (5%)	
2 (GS 3 + 4)	98 (38%)	63 (39%)	28 (38%)	7 (32%)	
3 (GS 4 + 3)	54 (21%)	26 (16%)	20 (27%)	8 (36%)	
4 (GS 8)	19 (7%)	7 (4%)	8 (11%)	4 (18%)	
5 (GS 9–10)	10 (4%)	3 (2%)	5 (7%)	2 (9%)	
Extraprostatic extension					<0.001
Absent	166 (64%)	125 (77%)	37 (51%)	4 (19%)	
Present	89 (34%)	37 (22%)	35 (48%)	17 (81%)	
Missing	4 (2%)	2 (1%)	1 (1%)	0	
Surgical margins					0.032
Negative	165 (64%)	113 (69%)	38 (52%)	14 (64%)	
Positive	92 (36%)	49 (30%)	35 (48%)	8 (36%)	
Missing	2 (1%)	2 (1%)	0	0	
Seminal vesicle invasion					<0.001
Absent	228 (88%)	157 (96%)	60 (83%)	11 (50%)	
Present	30 (12%)	7 (4%)	12 (16%)	11 (50%)	
Missing	1 (0%)	0	1 (1%)	0	
Lymph node involvement					0.001
Absent	252 (97%)	164 (100%)	68 (93%)	20 (91%)	
Present	7 (3%)	0	5 (7%)	2 (9%)	
CAPRA-S risk group ^†^					<0.001
Low	78 (30%)	62 (38%)	13 (18%)	3 (14%)	
Intermediate	113 (44%)	79 (48%)	29 (40%)	5 (23%)	
High	60 (23%)	19 (12%)	28 (38%)	13 (62%)	
Missing	8 (3%)	4 (2%)	3 (4%)	1 (1%)	
CAPRA-S risk group ^‡^					<0.001
Low	100 (39%)	77 (47%)	18 (25%)	5 (23%)	
Intermediate	93 (36%)	66 (40%)	23 (32%)	4 (18%)	
High	58 (22%)	17 (10%)	29 (40%)	12 (55%)	
Missing	8 (3%)	4 (2%)	3 (4%)	1 (5%)	

Data are median (Interquartile range (IQR)) or n (%). CAPRA-S = Cancer of the Prostate Risk Assessment Postsurgical; GS = Gleason score; PSA = prostate-specific antigen; PTEN = phosphatase and tensin homolog. * Fisher’s exact (categorical variables) or Kruskal–Wallis H (continuous variables) test were used to evaluate associations. ^†^ Assessed using centrally reviewed Gleason scores. ^‡^ Assessed using routine Gleason scores.

**Table 2:** Uni- and multivariable analyses of time to biochemical recurrence including Gleason grade groups assessed using routine Gleason scores.

Variable	Group	Univariable Analysis		Multivariable Analysis *	
		HR (95% CI)	*p* Value	HR (95% CI)	*p* Value
(A) Standard clinicopathologic parameters					
Ploidy and PTEN status			<0.0001		0.017
	Diploid and PTEN-high	ref.		ref.	
	Non-diploid or PTEN-low	1.94 (1.15–3.30)		1.11 (0.62–1.99)	
	Non-diploid and PTEN-low	4.63 (2.50–8.57)		2.82 (1.34–5.94)	
Age at surgery	10-year increment	1.51 (1.00–2.26)	0.048	0.98 (0.94–1.03)	0.51
Preoperative PSA	log_2_(1 + ng/mL) increment	2.39 (1.74–3.28)	<0.0001	2.44 (1.52–3.91)	<0.0001
Gleason grade group			<0.0001		0.017
	1 (GS 6)	ref.		ref.	
	2 (GS 3 + 4)	8.27 (2.52–27.17)		3.97 (1.17–13.48)	
	3 (GS 4 + 3)	10.25 (3.02–34.79)		3.86 (1.07–13.88)	
	4 (GS 8)	22.91 (6.38–82.24)		6.51 (1.65–25.72)	
	5 (GS 9–10)	57.17 (15.64–208.92)		10.08 (2.43–41.79)	
Extracapsular extension	Present vs. Absent	3.97 (2.45–6.44)	<0.0001	1.52 (0.84–2.75)	0.17
Surgical margins	Positive vs. Negative	2.90 (1.80–4.67)	<0.0001	2.09 (1.21–3.59)	0.008
Seminal vesicle invasion	Present vs. Absent	5.39 (3.22–9.02)	<0.0001	1.64 (0.88–3.06)	0.12
Lymph node involvement	Present vs. Absent	6.25 (2.67–14.60)	<0.0001	2.33 (0.89–6.14)	0.09
(B) CAPRA-S risk groups					
Ploidy and PTEN status			<0.0001		0.011
	Diploid and PTEN-high	ref.		ref.	
	Non-diploid or PTEN-low	1.94 (1.15–3.30)		1.19 (0.68–2.07)	
	Non-diploid and PTEN-low	4.63 (2.50–8.57)		2.69 (1.39–5.21)	
CAPRA-S risk group					
	Low (score 0–2)	ref	<0.0001	ref.	<0.0001
	Intermediate (score 3–5)	4.52 (1.85–11.01)		4.63 (1.89–11.31)	
	High (score ≥ 6)	17.44 (7.36–41.35)		14.68 (6.07–35.52)	

CAPRA-S = Cancer of the Prostate Risk Assessment Postsurgical; CI = confidence interval; GS = Gleason score; HR = hazard ratio; PSA = prostate-specific antigen; PTEN = phosphatase and tensin homolog. * Of the 259 patients included in univariable analyses (71 with event and 188 without event), 251 (69 with event and 182 without event) had complete data and were included in the multivariable analysis.

## Data Availability

The datasets analyzed during the current study are not publicly available due to the use of dis-identified (not anonymous) data but are available from the corresponding author on reasonable request.

## Supplementary Materials

The following are available online at https://www.mdpi.com/article/10.3390/cancers13174291/s1, Table S1: Clinicopathological characteristics and follow-up for all patients in the test subset and separately for patients with PTEN-high and PTEN-low. Table S2: Uni- and multivariable analyses of time to biochemical recurrence including centrally reviewed Gleason scores. Figure S1: Kaplan–Meier analysis of time to biochemical recurrence after radical prostatectomy in the validation cohort stratified by PTEN status. Figure S2: Overview of analyzed patient material and applied methods in the validation cohort. Figure S3: Kaplan–Meier analysis of time to biochemical recurrence after radical prostatectomy stratified by the automatically assessed PTEN status in the validation cohort. Figure S4: Kaplan–Meier analysis of time to biochemical recurrence after radical prostatectomy stratified by the automatically assessed PTEN status in the validation cohort. Figure S5: Kaplan–Meier analysis of time to biochemical recurrence after radical prostatectomy stratified by the combined automatically assessed PTEN and DNA ploidy status in the validation cohort. File S1: Independent validation in prostate cancer of the prognostic value of a deep learning system for assessment of phosphatase and tensin homologue (PTEN) status in immunohistochemically stained tissue slides.
