# Supplementary material for: PTEN and DNA Ploidy Status by Machine Learning in Prostate Cancer
Source: Cancers (Basel). 2021 Aug 26;13(17):4291. doi: 10.3390/cancers13174291 (PMC8428363; doi:10.3390/cancers13174291)
Supplement: Supplementary file 1 [file cancers-13-04291-s001.zip › PTEN and DNA ploidy status by machine learning in prostate cancer_protocol.pdf]

Independent validation in prostate cancer of the prognostic value of a deep learning system for assessment of phosphatase and tensin homologue (PTEN) status in immunohistochemically stained tissue slides

1. Status at last amended

This protocol was last modified on 28th of October 2020, prior to all investigations that could reveal associations between PTEN status and clinical outcome (i.e. biochemical recurrence) in the validation cohort. At that time the immunohistochemically stained tissue slides from the validation cohort had been scanned and tiled blinded to the clinical outcome.

2. Patients and specimens

A discovery cohort and an independent validation cohort were included in this study. Both cohorts comprised patients operated for primary prostate cancer at the Norwegian Radium Hospital, Oslo; a tertiary comprehensive cancer center in Norway. The basis for selection of patients to radical prostatectomy (RP) was preoperative absence of known metastasis, age less than 75 years and life expectancy of at least 10 years. Patients from the discovery cohort were operated by one surgeon, and patients from the validation cohort were operated by another surgeon. Each prostate gland was processed into a series of 3–5 mm thick formalin-fixed, paraffin-embedded tissue blocks. In each cohort, biomarker status was assessed in three tumor-containing blocks to better represent prostate tumors in consideration of intratumor heterogeneity [1].

2.1 Discovery cohort

The discovery cohort comprised 317 patients who underwent RP between 1987 and 2005, and were operated by one surgeon (HW). Ten patients were excluded due to: preoperative therapy (n = 1), death from postoperative complications (n = 1), loss to follow-up (n = 1) or lack of available tumor material (n = 7) (Protocol Fig. 1). Out of the 307 eligible patients, we selected a subset of 253 patients with three available tumor-containing blocks, based on the highest Gleason sum and/or previously assessed non-diploid DNA ploidy status [1]. Among the other 54 patients, 38 patients had an available tumor-containing block representing the highest Gleason score, and this subset was used for training and tuning of the PTEN classifier. For training and tuning of the tumor detector, the subset of 38 patients was supplemented with the three available tumor-containing blocks from 50 randomly selected patients from the subset of 253 patients and 20 tumor-containing blocks from ten patients not included in the subset (four of these ten patients were included in the subset of the 38 patients). The subset of 253 patients was used as a test set for comparing the performance of the automatic PTEN scoring (involving both automatic tumor detection and automatic PTEN classification) and the visual PTEN scoring method, as well as for selecting an optimal threshold for dichotomizing the fraction of PTEN positive tumor cells.

Neoadjuvant therapy was not given to any of the patients included in the test subset. Adjuvant radiotherapy and/or hormone treatment was not routinely applied, but patients were offered radiotherapy and/or hormone treatment after indication of recurrence.

New tissue sections were cut for this study, hematoxylin and eosin (H&E) stained and reviewed by a pathologist (MP) who marked tumor areas >4 mm^2 on each slide. Tumor area was defined as a continuous region of prostate carcinoma. If multiple tumor areas were present in a tissue section, they were considered as independent tumor areas if they were situated ≥3 mm apart. One 3 µm tissue section was cut for PTEN immunohistochemistry (IHC) from each tumor-containing tissue block. One to three 50 µm tissue sections were macrodissected for DNA ploidy analysis by image cytometry from each tumor area. When preparing monolayers (see Section 3.3), the macrodissected tumor areas were treated individually, i.e. a separate monolayer was made from each tumor area, while PTEN was assessed for each entire slide.

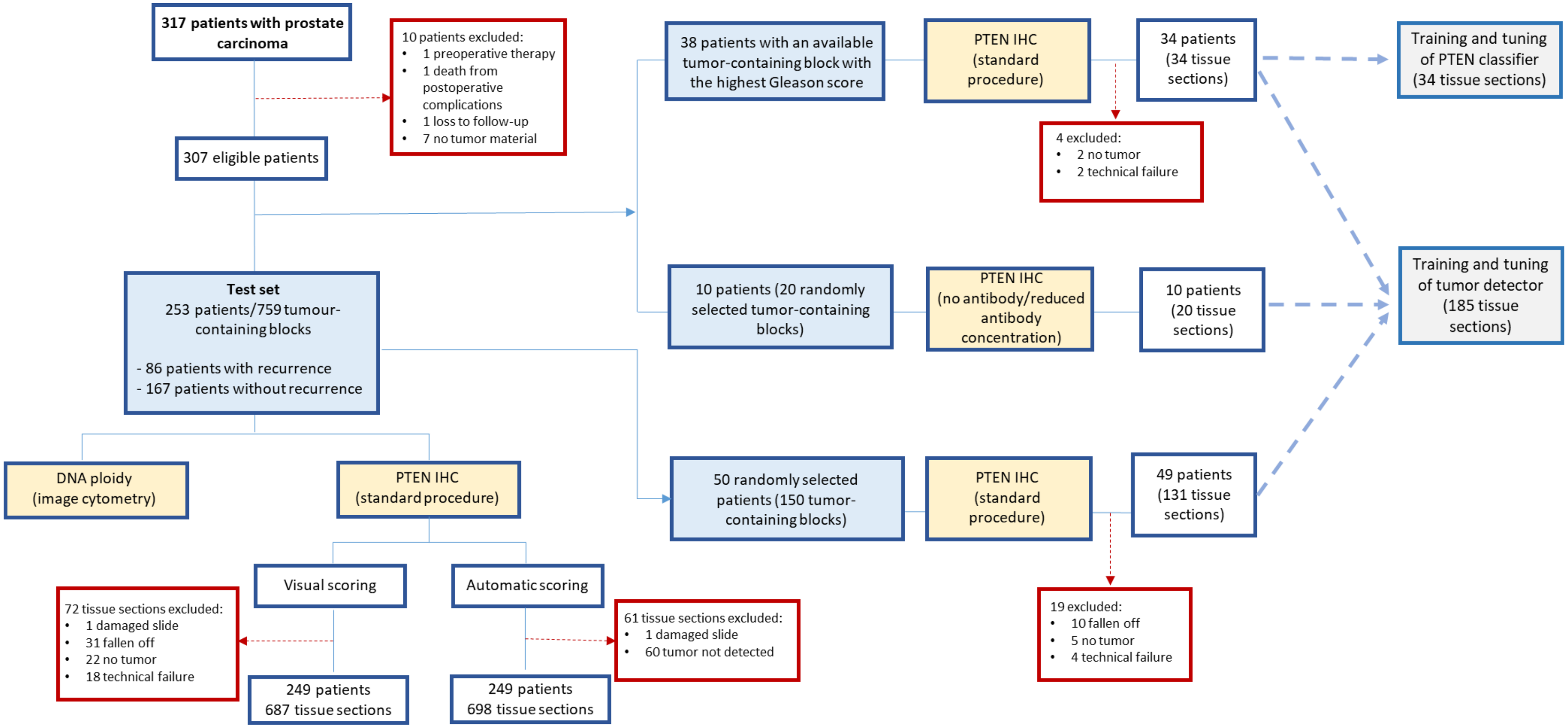

Protocol Fig. 1: A diagram specifying inclusions and exclusions of patients, tumor tissue blocks and tissue sections from the discovery cohort, as well as datasets used for training and tuning of PTEN classifier and tumor detector. Out of the 307 eligible patients, we selected a subset of 253 patients with three available tumor tissue blocks with the highest Gleason sum and/or previously assessed non-diploid DNA ploidy status for each patient. PTEN classifier was trained and tuned using 38 patients with one available tumor-containing block with highest Gleason score and that were not included in the subset of 253 patients, of whom 34 had valid PTEN slides. For training and tuning of the tumor detector, this subset of 34 patients was supplemented by PTEN IHC-stained tissue sections from 50 randomly selected patients included in the subset of 253 patients, of whom 49 had valid PTEN slides. In addition, we included 20 tumor-containing blocks from 10 patients not included in the subset of 253 patients (four of these ten patients were included in the subset of the 38 patients), for which PTEN IHC staining was performed without antibody or with antibody concentration 3x more diluted in order to obtain cases with technical failures.

Abbreviations: IHC = immunohistochemistry, PTEN = phosphatase and tensin homologue.

2.2 Validation cohort

The validation cohort comprised 287 patients who underwent RP between 2001 and 2006, and were operated by one surgeon (BB). A total of 28 patients were excluded due to: missing consent (n = 21), missing or less than six weeks of follow-up (n = 4) or no tumor material (n = 3) (Protocol Fig. 2). We selected three tumor-containing blocks for each of the 259 eligible patients. The first and the second block represented the worst Gleason score and the largest tumor area, respectively. The third block was selected randomly from the remaining blocks with a tumor area >0.5 cm^2.

Two patients received neoadjuvant therapy and 16 patients received adjuvant hormonal or radiotherapy within the six first months after surgery. Therapy started more than 6 months after surgery was considered as secondary treatment.

New tissue sections were cut for this study, H&E stained and reviewed by a pathologist (MP) who marked tumor areas >5 mm^2 on each slide. Tumor area was defined as a continuous region of prostate carcinoma. One 3 µm tissue section was cut for PTEN IHC from each tumor-containing tissue block. One to three 50 µm tissue sections were macrodissected for DNA ploidy analysis by image cytometry from each tumor area. When preparing monolayers (see Section 3.3), the macrodissected tumor areas from each block were combined, and one monolayer was made from each block. Similarly, PTEN was assessed for each entire slide, not separately for the individual tumor areas.

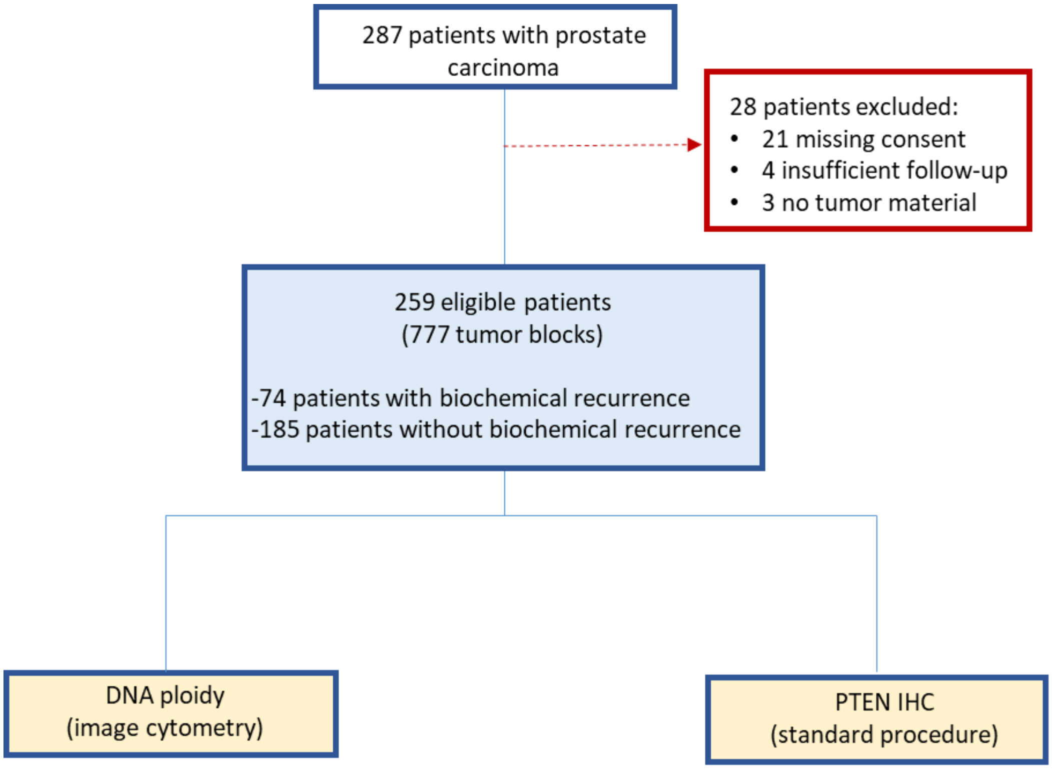

Protocol Fig. 2: A diagram specifying inclusions and exclusions of patients and tumor tissue blocks from the validation cohort.

Abbreviations: IHC = immunohistochemistry, PTEN = phosphatase and tensin homologue.

3. Methods

3.1 Gleason scoring

All available routine histological sections from both cohorts were centrally reviewed by an experienced uropathologist (LV). Gleason scoring in the discovery cohort was performed according to the updated 2005 International Society of Urological Pathology (ISUP) consensus guidelines [2, 3], whereas in in the validation cohort, it was performed according to the 2014 ISUP consensus guidelines [4]. Gleason scores in both cohorts were arranged into the five prognostic Gleason grade groups (GGG) following the 2014 ISUP consensus guidelines [4], i.e., GGG 1 if Gleason score 3 + 3 = 6, GGG 2 if Gleason score 3 + 4 = 7, GGG 3 if Gleason score 4 + 3 = 7, GGG 4 if Gleason score 3 + 5 = 8, Gleason score 5 + 3 = 8 or Gleason score 4 + 4 = 8, and GGG 5 if Gleason score 4 + 5 = 9, Gleason score 5 + 4 = 9 or Gleason score 5 + 5 = 10.

3.2 Immunohistochemistry, scanning of tissue slides and visual PTEN scoring

PTEN immunohistochemistry was performed on 3 μm tissue sections using the Envison FLEX+ system/Dako Autostainer Link 48 (Agilent Technologies, Santa Clara, CA). Deparaffinization and unmasking of epitopes were performed using EnVisionTM Flex Target Retrieval Solution at 97ºC for 20 min. Endogenous peroxidase was blocked by treating the sections with FLEX peroxidase-blocking reagent for 5 min. Further, the tissue sections were incubated for 120 min with the rabbit monoclonal PTEN antibody (1:400, 138G6, Cell Signaling Technology, Danvers, MA, USA), followed by EnVision FLEX+ Rabbit (linker) for 15 min, EnVisionTM Flex/HRP enzyme for 20 min and 3,3'-diaminobenzidine tetrahydrochloride chromogen for 10 min. Finally, the tissue sections were dehydrated and counterstained with hematoxylin for 10 seconds.

All tissue sections were scanned on NanoZoomer XR digital slide scanner (Hamamatsu Photonics, Hamamatsu, Japan), at the highest resolution available (termed 40x). The resulting whole-slide images (WSIs) had pixels each representing a physical size of 0.227 μm both vertically and horizontally. Tissue sections from the discovery cohort and the validation cohort were processed in the same manner.

PTEN expression was visually scored at 10%-intervals, i.e. 0%, (0%, 10%], (10%, 20%], ..., (80%, 90%] and (90%, 100%], by two trained experts (KC and EE), blinded to clinical and outcome data. Benign glands and/or stroma served as internal positive controls. PTEN expression was not scored

when the intensity of the staining was weak or absent in both the tumor cells and internal positive controls (ambiguous staining) or when ≥95% of the tumor area had fallen off during the IHC procedure. Scoring was performed using two scoring protocols, which differently defined positive and negative PTEN protein expression. In scoring protocol 1, tumor cells were considered PTEN positive when cytoplasmic and/or nuclear staining was present (Protocol Fig. 3A-3B), whereas tumor cells with markedly reduced staining intensity compared with adjacent PTEN positive tumor glands or benign glands (Protocol Fig. 3C-3D) or absent cytoplasmic and/or nuclear staining (Protocol Fig. 3E-3F) were considered PTEN negative, as described previously [5, 6]. In scoring protocol 2, tumor cells were considered PTEN positive when cytoplasmic and/or nuclear staining was present (Protocol Fig. 3A-3D), also when staining intensity was markedly reduced compared with adjacent PTEN positive tumor glands or benign glands, whereas tumor cells with absent cytoplasmic and/or nuclear staining (Protocol Fig. 3E-3F) were considered PTEN negative, as described previously [7]. Discordant cases were discussed and a consensus PTEN score was reached for each case, which was the score used in further analyses.

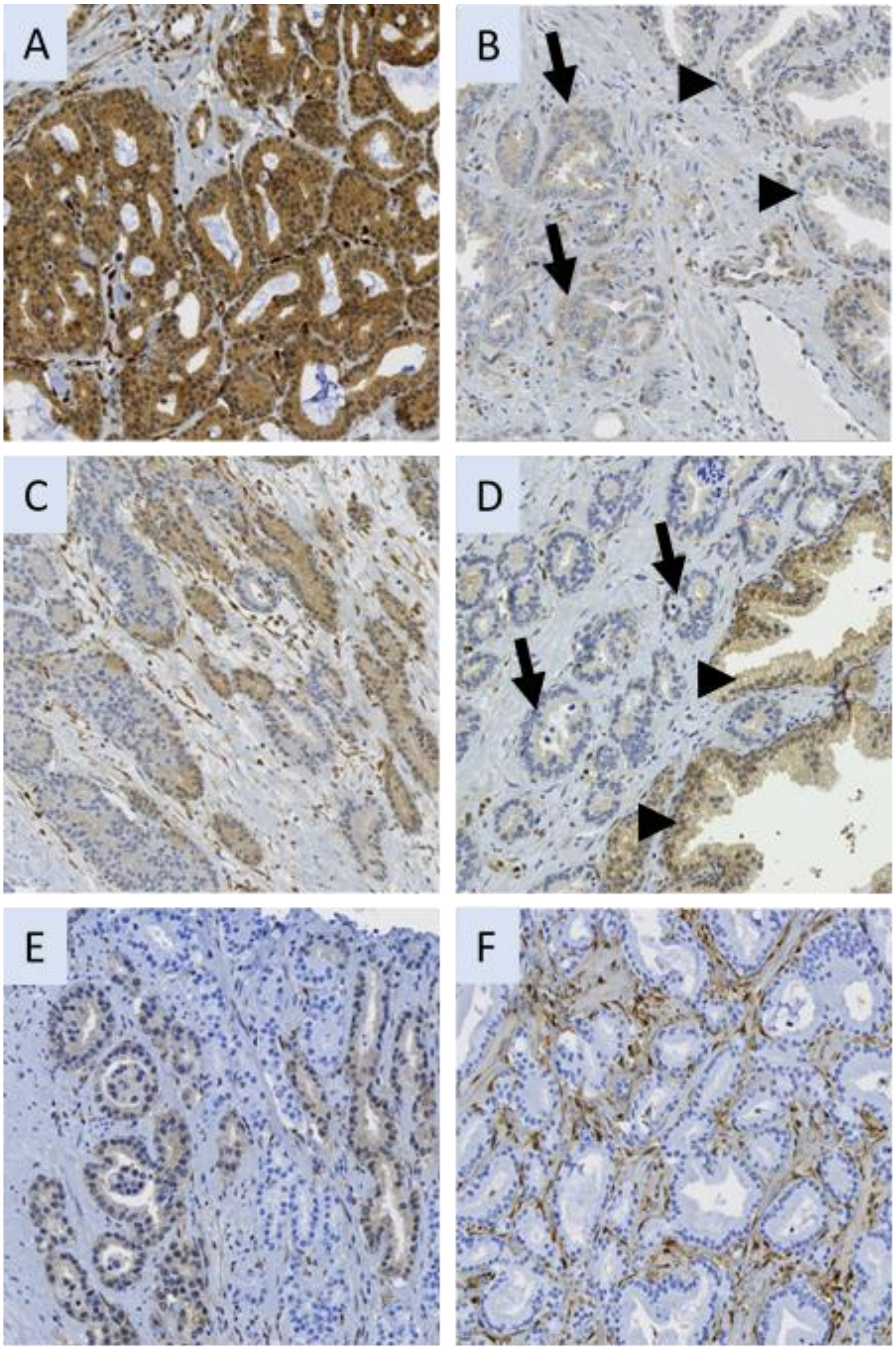

Protocol Fig. 3: Types of phosphatase and tensin homologue (PTEN) immunostaining. **(A)** Strong PTEN staining present. **(B)** Weak PTEN staining present in tumor glands (arrows) and benign glands (arrowheads). **(C)** Reduced PTEN staining in a fraction of tumor cells. **(D)** Reduced PTEN staining in tumor glands (arrows) compared to cells in benign glands. **(E)** PTEN staining absent in both nucleus and cytoplasm in a fraction of tumor cells. **(F)** PTEN staining absent in both nucleus and cytoplasm in all tumor cells.

3.3 DNA ploidy by image cytometry

Preparation of nuclear monolayers was performed on 50 µm thick, macrodissected tumor areas according to a modified Hedley's method [8]. Briefly, the 50µm tissue sections were deparaffinized using xylene and rehydrated with a series of decreasing concentrations of ethanol, followed by a rinse in phosphate buffered saline. The isolation of single nuclei was done by enzymatic digestion (using protease from *Bacillus licheniformis* Type VIII [P8038], Sigma Chemical, St Louis, MO, USA) and mechanical disruption of the tissue (using magnetic stirring). Next, the suspension was filtered through a 60 µm mesh nylon filter to remove larger fragments of undigested tissue. The filtrate was pipetted into a cytospin chamber and centrifuged in a cytospin centrifuge (Cytospin4, Thermo Scientific (Waltham, MA, USA)) for 5 min at 600 rpm onto a poly-l-lysine-coated glass slide. The resulting slides with nuclear monolayers were fixed in 4% buffered formalin, followed by DNA-specific staining by the Feulgen method. The Feulgen staining was performed by incubating the slides in 5 M hydrochloric acid for 60 min at room temperature for hydrolysis, followed by staining with Schiff's solution for 2 h in the dark and rinsing three times for 10 minutes in a freshly-prepared solution of 0.5% sodium metabisulfite in 0.05 M hydrochloric acid. Finally, the slides were dehydrated with a series of increasing concentrations of ethanol, immersed in xylene and coverslipped. Feulgen-stained nuclei were imaged by a Zeiss AxioImager microscope (AxioImager A1/A2 brightfield microscope, Zeiss, Germany) equipped with a 546 nm green filter and a 40x lens with a numerical aperture of 0.75. A high-resolution camera (AxioCam MrM, Zeiss, Germany) connected to the microscope was used to acquire digital images. Nuclei in the images were segmented from the background by the Ploidy Work Station (PWS) Grabber software (Room4 Ltd, Sussex, UK). The complete procedure for performing DNA ploidy by image cytometry is published as a video on YouTube [9].

Identification of representative epithelial and reference nuclei, and DNA ploidy histogram classification into diploid, tetraploid or aneuploid was done automatically using the PWS Classifier software (Room4 Ltd, Sussex, UK). A sample was classified as diploid when only one 2c peak was present and when the only other peak was a 4c peak containing at most 15% of the total number of nuclei, in both cases additionally requiring that less than 1% of the total number of nuclei had DNA content exceeding 5c. A sample was classified as tetraploid when only a 4c peak was present in addition to the 2c peak and the number of nuclei in the 4c peak exceeded 15% of the total number of nuclei. Samples with an 8c peak were also classified as tetraploid when

otherwise only a 2c peak and a 4c peak was present. A sample was classified as aneuploid when a peak was present outside of 2c, 4c or 8c areas and when there was at least 1% non-euploid nuclei with a DNA content exceeding 5c among the total number of nuclei. Samples with less than 300 nuclei were considered indeterminate. A trained expert reviewed the automatic histogram classifications. In case of contradictory interpretation of a histogram, a consensus classification was made by consulting with another expert (HED), and this consensus was used in further analyses.

DNA ploidy classifications obtained from all analyzed blocks for each patient were compiled, and the DNA ploidy classification that indicated the worst prognosis for each patient was used in further analyses. Aneuploidy was considered the DNA ploidy classification indicating the worst prognosis, followed by tetraploid, whereas diploid samples were considered to indicate good prognosis [10]. For all analysis, patients were categorized as diploid or non-diploid, where the latter category contained tumors classified as either tetraploid and aneuploid.

Results on DNA ploidy classification in the test subset and the validation cohort were published previously [11]. After this publication we have replaced some of the tumor-containing tissue blocks in the test subset and the validation cohort, which have resulted in different DNA ploidy classification for three patients and one patient, respectively. In the test subset, 57 tumor-containing tissue blocks in the test subset, as they did not contain sufficient tissue material for this study. In the validation cohort, one of the tumor-containing tissue blocks in the validation cohort was replaced as we discovered that it did not contain prostate adenocarcinoma but bladder urothelial carcinoma.

3.4 Statistical analyses

Statistical calculations were performed using Stata/MP 16.1 (StataCorp, College Station, TX, USA) and SPSS (v26.0, IBM Corporation, Armonk, NY, USA). Correlations between the automatic and the visual PTEN scores were evaluated using Pearson correlation coefficient.

In the survival analyses of the test set, we used recurrence, biochemical recurrence (BCR), metastases and cancer-specific survival as endpoints. Recurrence was defined, in accordance with Punt *et al*. [12], as locoregional recurrence (confirmed by histological biopsies or ultrasound), distant metastasis (detected by skeletal scintigraphy) or death from prostate cancer (based on death

certificate). BCR was defined as a single prostate-specific antigen (PSA) ≥0.4 ng/ml. Time to event (recurrence, BCR, metastasis or cancer-specific death) was calculated from primary surgery to event (recurrence of disease, BCR, metastasis of disease or cancer-specific death, respectively), non-related death or the last date of follow-up (31st of December 2008), whichever occurred first.

BCR, defined as a single PSA ≥0.4 ng/ml, was used as an endpoint in survival analyses of the validation cohort. Time to BCR was calculated from surgery to the onset of the BCR event in question or to the date of the last recorded PSA measurement (24th of June 2020). PSA measurements within 6 weeks after surgery were not considered when identifying BCR.

Univariable survival analyses were performed using the Kaplan-Meier method, and survival curves were compared with the log-rank test. Cox proportional hazards regression analysis was performed to test the statistical independence and significance between pathological, molecular and clinical variables. The following variables were included in the multivariable model: age (continuous), PSA level (log_2 transformed), GGG (categorical) and the dichotomous pathologic staging parameters extraprostatic extension (EPE), surgical margin (SM), seminal vesicle invasion (SVI) and lymph node invasion (LNI). The Cancer of the Prostate Risk Assessment (CAPRA-S) score was calculated using preoperative PSA, Gleason score and the four pathologic staging parameters, and was grouped to stratify patients into low (score 0 to 2), intermediate (score 3 to 5), and high (score ≥6) risk groups, as described previously [13]. Harrell's concordance index (c-index) [14] was used to report the studied markers' ability to predict outcome. The confidence interval (CI) of the c-index was computed as the bias-corrected and accelerated (BCa) percentile interval over 10,000 bootstraps [15]. DNA ploidy and PTEN status were integrated with the CAPRA-S score by adding 1 point if non-diploid and 1 point if PTEN loss. C-indices were calculated for the updated CAPRA-S score based on the score without categorization into three risk groups. Two-sided p-value for test of difference in c-index between the standard and the updated CAPRA-S score was calculated as 1 minus the confidence level of the largest BCa CI that did not contain 0. Two-sided p-values <0.05 were considered statistically significant.

4. Automatic PTEN scoring

4.1 PTEN classifier

4.1.1 Dataset, manual annotations and tiling

One tumor-containing block with highest Gleason score was selected for each of the 38 patients not included in the subset of 253 patients (Protocol Fig. 1). From each block, a 3 µm section was cut, IHC-stained for PTEN and scanned as described in Section 3.2. Of the resulting 38 WSIs, four were excluded due to: no tumor (n=2) or a technical failure (n=2). Tumor areas were manually annotated in the 34 WSIs of the valid PTEN IHC slides by a trained expert (KC), based on the annotation of tumor in parallel H&E scans performed by a pathologist (MP). The annotated tumor areas were partitioned into non-overlapping regions of fixed size, called tiles. The tile size was 1024x1024 pixels, which corresponds to 232.45 µm both vertically and horizontally in the original tissue slide. Manual annotations and tiling were performed using a ImmunoPath tool (Room4 Ltd, Sussex, UK).

A total of 10 tumor tiles were randomly selected from each of the 34 WSIs. Within the selected tiles, PTEN positive and PTEN negative tumor nuclei were exhaustively annotated by the trained expert (KC) using a NLine tool (Institute for Cancer Genetics and Informatics (ICGI), Oslo University Hospital (OUH), Norway). The annotations were made manually by drawing the contours of the nuclei; separate colors were used to label PTEN positive nuclei and PTEN negative nuclei. Nuclei were labelled as PTEN positive or PTEN negative according to scoring protocol 2 (see Section 3.2 for details). We could not reliably apply scoring protocol 1 because it requires comparing of staining intensity of tumor glands to adjacent benign glands (or other PTEN positive tumor glands) in order to determine whether the staining is weak (Protocol Fig. 3B) or "markedly reduced" (Protocol Fig. 3C–3D), but such controls are not always available when evaluating the small tumor regions visible in a tile. Also, since reduced staining is uncommon and was not observed in the set of tiles from the 34 WSIs, the use of scoring protocol 2 instead of scoring protocol 1 appears to be of minor importance.

For each annotated tile, the corresponding image region in the WSI was identified and extracted at full resolution (termed 40x), and the annotations of tumor nuclei were scaled to the same resolution (by upsampling with a factor of 2.584). New tiles with 800x800 pixels were generated,

starting at the upper left corner, resulting in nine adjacent, non-overlapping tiles for each extracted image region of the WSI with corresponding tumor nuclei annotations. These tiles were used as the ground truth in training and tuning of the PTEN classifier, and were randomly split on patient level into a train subset containing 24 WSIs (70%) and a tune subset containing 10 WSIs (30%). Empty tiles, i.e. tiles without any annotations of either PTEN positive nuclei or PTEN negative nuclei, were not included in the training but were included for evaluation of the trained models.

4.1.2 Training and tuning of the PTEN classifier

An instance segmentation neural network was trained to detect and delineate tumor cells and classify them as either PTEN positive or PTEN negative. The input and target output during network training were the tiles with 800x800 pixels and the associated manual PTEN positive and PTEN negative tumor nuclei annotations.

The instance segmentation network used was MaskRCNN [16], which is a region-based convolutional neural network that outputs a segmentation and a class label for each detected object. It works in two stages. The first stage generates object proposals in the shape of bounding boxes, i.e. rectangular regions where it is found probable that a target object in the input image might be. The second stage predicts the class label of the proposed object, refines the bounding box and generates a segmentation mask on pixel level for the object. Both of these stages are connected to a backbone structure that is a convolutional neural network; we used ResNet-50-FPN (FPN=Feature Pyramid Network).

Models were trained using NVIDIA's implementation of Mask RCNN in the NVIDIA container image for PyTorch release 19.12 (https://docs.nvidia.com/deeplearning/frameworks/pytorch-release-notes/rel_19-12.html#rel_19-12). Default hyperparameters were used unless otherwise specified below. Trainings were performed on a machine with 4 NVIDIA Titan Xp GPU cards using a batch size of 8 tile images, i.e. 2 tile images per GPU. The tile sampling was performed randomly on the tile level.

Before inputted to the network during training, each tile was distorted. All distortions were done using torchvision transforms (https://pytorch.org/docs/stable/torchvision/transforms.html). The following distortions were performed in the listed order:

1. The image was flipped horizontally with a probability of 0.5, or remained unflipped (also with a probability of 0.5).
2. The image was converted from red, green and blue (RGB) color space to hue, saturation and value (HSV) color space, the hue channel was cyclically shifted with a random value drawn from the uniform distribution on [-0.1, 0.1], and the image was converted back to RGB color space, all using the function torchvision.transforms.functional.adjust_hue. For reference, note that a cyclical shift of -0.5 or 0.5 gives an image with complementary colors.
3. Saturation was adjusted with a saturation factor randomly drawn from the uniform distribution on [0.8, 1.2] using the function torchvision.transforms.functional.adjust_saturation.
4. Brightness was adjusted with a brightness factor randomly drawn from the uniform distribution on [0.9, 1.1] using the function torchvision.transforms.functional.adjust_brightness.
5. Contrast was adjusted with a contrast factor randomly drawn from the uniform distribution [0.8, 1.2] using the function torchvision.transforms.functional.adjust_contrast.

Each pixel in the randomly distorted image was then standardized by subtracting 122.7717 from red color channel, 115.9465 from green color channel, and 102.9801 from the blue color channel.

The ResNet-50-FPN backbone was pretrained on ImageNet (https://docs.nvidia.com/deeplearning/frameworks/pytorch-release-notes/rel_19-12.html#rel_19-12). In our training, the first bottom 2 layers were frozen. We used FPN in both the region proposal network (RPN) and in the region of interest (ROI) Heads. For the region proposal network, the anchor size and anchor strides were both set to (4, 8, 16, 32, 64) pixels of the input tiles with 800x800 pixels. An object proposal was considered a true object if the Intersection over Union (IoU) with any ground truth object was at least 0.7, and a background object if the IoU was maximum 0.3. The number of top scoring object proposals to keep before non-maximum

suppression (NMS) was set to 2000 per input tile image and, after applying NMS, at most 1000 object proposals were kept. The NMS threshold used for the object proposals was 0.7, i.e. the maximum allowed intersection over union for object proposals was 0.7.

In training the part of the network that performs bounding box regression, mask segmentation and object classification, an ROI was considered foreground if the IoU with any ground truth object was at least 0.5, and background otherwise. We used a batch size of 512 objects per image, which made the total number of ROIs per training minibatch = batch_size_per_image * images_per_gpu * number_of_gpus = 512 * 2 * 4 = 4096. The target fraction of ROIs labeled foreground was 0.25.

For the bounding box regression arm, of the network we used "FPN2MLPFeatureExtractor" and "FPNPredictor", and the following parameters: a pooler resolution of 7, pooler scales of (0.25, 0.125, 0.0625, 0.03125) and a pooler sampling ratio of 2. For the mask arm of the network, we used "MaskRCNNFPNFeatureExtractor" and "MaskRCNNC4Predictor", and the following parameters: a pooler resolution of 14, a pooler sampling ratio of 2, pooler scales of (0.25, 0.125, 0.0625, 0.03125) and a resolution of 28. The classification arm of the network outputs a score per class which reflects the probability of that class, and the class with maximum score is chosen as the predicted class.

The network was trained end-to-end using stochastic gradient descent. We used a momentum of 0.9 and gamma of 0.1. A linear warmup was used with 500 warmup-iterations and a warmup factor of 1/3. The base learning rate was 0.005, and the weight decay was 0.0001. Training was ceased at a specified iteration. Model performance was evaluated at different iterations to find the best model.

When using the trained network to run inference on a new case, the score threshold is set to 0.5, implying that all detections with a maximum class score less than 0.5 will be classified as background. NMS will be used during inference to suppress highly overlapping boxes; the number of the NMS threshold is then set to 0.5. The number of top scoring object proposals to keep before NMS was set to 2000 per input tile image. The maximum number of object detections per image during inference is set to 500. The learning rate was reduced 1/10 of previous value at these iteration steps in training.

4.1.3 PTEN classification models

First, a model identified as 200420 was trained using the 800x800 pixels tile images and three output classes: manually annotated PTEN positive tumor nuclei, manually annotated PTEN negative tumor nuclei and background (no manual annotation). Then, images were made with tile images overlaid with the manual annotations as well as automatic detections from the 200420 model that did not overlap with any of the manual annotations (IoU = 0). Different colors were used for the overlaid depending on whether the annotation was a manually annotated PTEN positive tumor nuclei, a manually annotated PTEN negative tumor nuclei, a predicted PTEN positive tumor nuclei, or a predicted PTEN negative tumor nuclei, as depicted in Protocol Fig. 4A and 4C. The trained expert (KC) reviewed images from the train and the tune subsets and reclassified predicted PTEN positive tumor nuclei to true PTEN positive tumor nuclei and predicted PTEN negative tumor nuclei to true PTEN negative tumor nuclei if the automatic detections were actually true (i.e. tumor nuclei with correct PTEN positive or PTEN negative label). Reclassification was done using a Manual Counter tool (ICGI, OUH, Norway) by setting a "+" sign for true PTEN positive tumor nuclei and a "-" sign for true PTEN negative tumor nuclei inside nuclei (Protocol Fig. 4B and 4D). Not reclassified predicted PTEN positive tumor nuclei constituted a new class termed false PTEN positive tumor nuclei, and not reclassified predicted PTEN negative tumor nuclei a new class termed false PTEN negative tumor nuclei. The predicted PTEN positive and PTEN negative tumor nuclei overlapping with a manual annotation were not included in the classes for false PTEN positive and false PTEN negative tumor nuclei. The manually annotated PTEN positive and PTEN negative tumor nuclei were included in the classes for true PTEN positive and true PTEN negative tumor nuclei. A new model, identified as 200423, was then trained with the same tile images (as for the 200420 model) and five output classes: true PTEN positive tumor nucleus, true PTEN negative tumor nucleus, false PTEN positive tumor nucleus, false PTEN negative tumor nucleus and background.

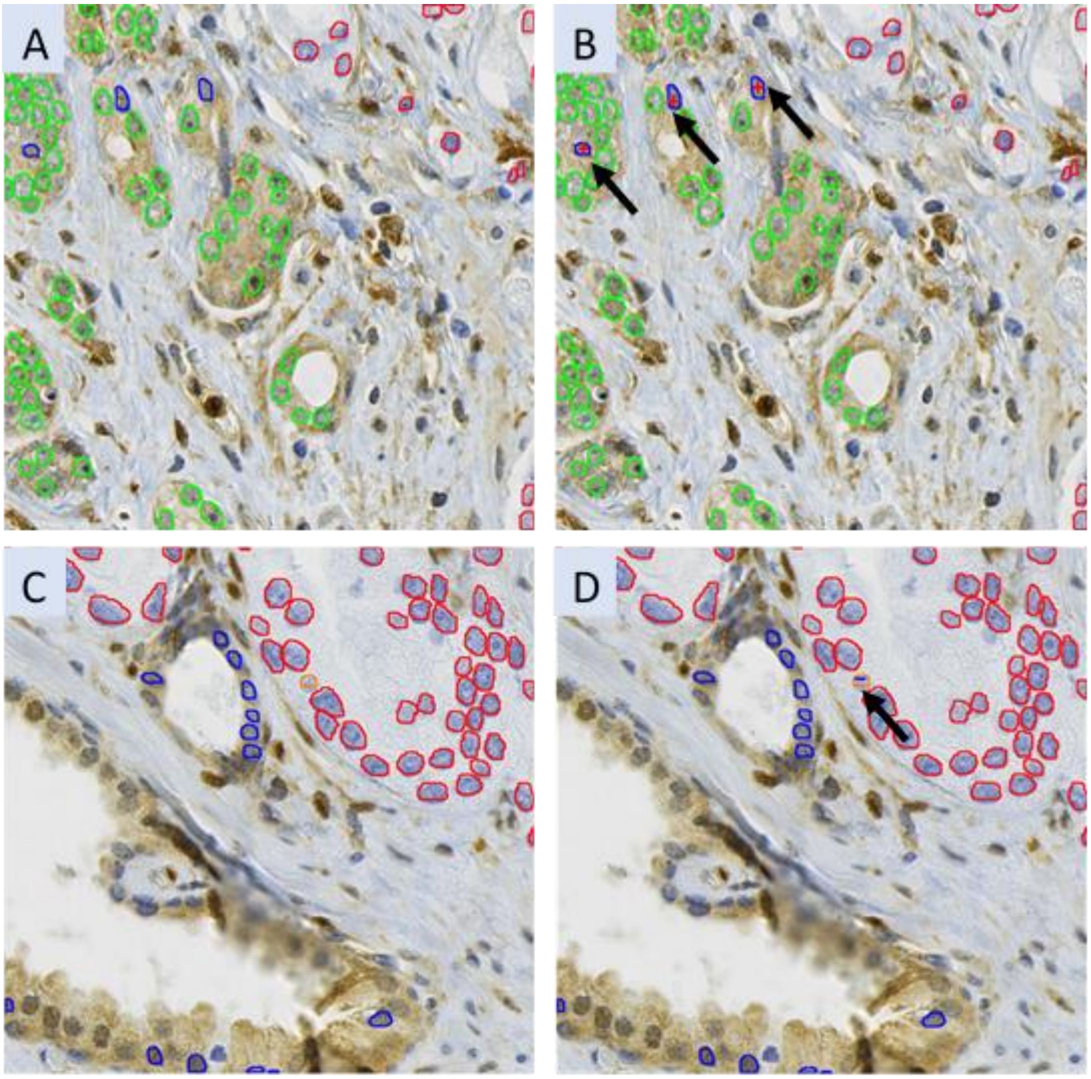

Protocol Fig. 4: Examples of tile images with manual annotations and non-overlapping automatic detections of PTEN positive and PTEN negative tumor nuclei, where the 200420 model was used to obtain the automatic detections. Manually annotated PTEN positive tumor nuclei are depicted as green, manually annotated PTEN negative tumor nuclei as red, non-overlapping predicted PTEN positive tumor nuclei as blue, and non-overlapping predicted PTEN negative tumor nuclei as orange. (A, C) Images reviewed by the trained expert. (B, D) Images resulting from the review, identifying the predicted PTEN positive tumor nuclei reclassified to true PTEN positive tumor nuclei as "+" and predicted PTEN negative tumor nuclei reclassified to true PTEN negative tumor nuclei as "–", overlaid with black arrows to mark reclassified nuclei.

Abbreviation: PTEN = phosphatase and tensin homologue.

Protocol table 1 and Protocol table 2 show the performance of the 200420 model and the 200423 model compared to its respective the ground truth. An automatic detection was considered correct if and only if the IoU with a ground truth object was more than 0.5 and the predicted class was the same as the ground truth class. Recall, precision and mean average precision (mAP) were then calculated using only the classes for true PTEN positive and true PTEN negative tumor nuclei, i.e. the classes for false PTEN positive and false PTEN negative tumor nuclei were ignored even for the 200423 model that predict them (similarly, the background class was also ignored, as is the convention when computing recall, precision and mAP). In the below specification of recall, precision and mAP, the performance is first provided for the tune subset of the selected tumor tiles from the 24 WSIs and then in parenthesis for the train subset of the selected tumor tiles from the 10 WSIs.

The 200420 model and the 200423 model were then applied on all tiles within the manually annotated tumor areas from the 34 WSIs. Scatterplots and Bland-Altman plots were made to assess correlation and agreement of the fraction of PTEN positive tumor cells (i.e. the PTEN scores) obtained by visual scoring compared to the 200420 model (Protocol Fig. 5) and the 200423 model (Protocol Fig. 6). The fraction of PTEN positive tumor cells for an automatic model was calculated as the ratio between the number of objects predicted to be true PTEN positive tumor nuclei and the total number of objects predicted to be either true PTEN positive or true PTEN negative tumor nuclei, thus ignoring predictions of false PTEN positive and false PTEN negative tumor nuclei (as well as background).

Model 200420

Dataset

Train subset:

- Number of tiles in dataset (including empty tiles): 2160
- Number of empty tiles in dataset: 947
- Number of tiles with at least one manually annotated PTEN positive tumor nucleus: 987
- Number of tiles with at least one manually annotated PTEN negative tumor nucleus: 316

Number of nuclei per class in train subset:

- Manually annotated PTEN positive tumor nuclei: 46434
- Manually annotated PTEN negative tumor nuclei: 11146

Tune subset:

- Number of tiles in dataset (including empty tiles): 900
- Number of empty tiles in dataset: 423
- Number of tiles with at least one manually annotated PTEN positive tumor nucleus: 437
- Number of tiles with at least one manually annotated PTEN negative tumor nucleus: 55

Number of nuclei per class in tune subset:

- Manually annotated PTEN positive tumor nuclei: 17396

Manually annotated PTEN negative tumor nuclei: 2801

Training:

- 3 classes: background (no manual annotation), manually annotated PTEN positive tumor nuclei and manually annotated PTEN negative tumor nuclei.
- Trained for 100000 iterations.
- Steps: (50000, 75000)

-

Evaluation in the tune subset and the train subset:

Recall: 0.699 (0.921)

Precision: 0.561 (0.691)

mAP: 0.687 (0.842)

Protocol table 1: Confusion matrix of the classification results on the tune subset using the 200420 model.

	Predicted PTEN positive tumor nuclei	*Predicted PTEN negative tumor nuclei*	*Ground truth object not found or classified as background*	*Number of ground truth objects*
No manual annotations	10151	692	NA	NA
Manually annotated PTEN positive tumor nuclei	11798	32	5724	17396
Manually annotated PTEN negative tumor nuclei	5	2506	316	2801
Total	21954	3230	6040	20197

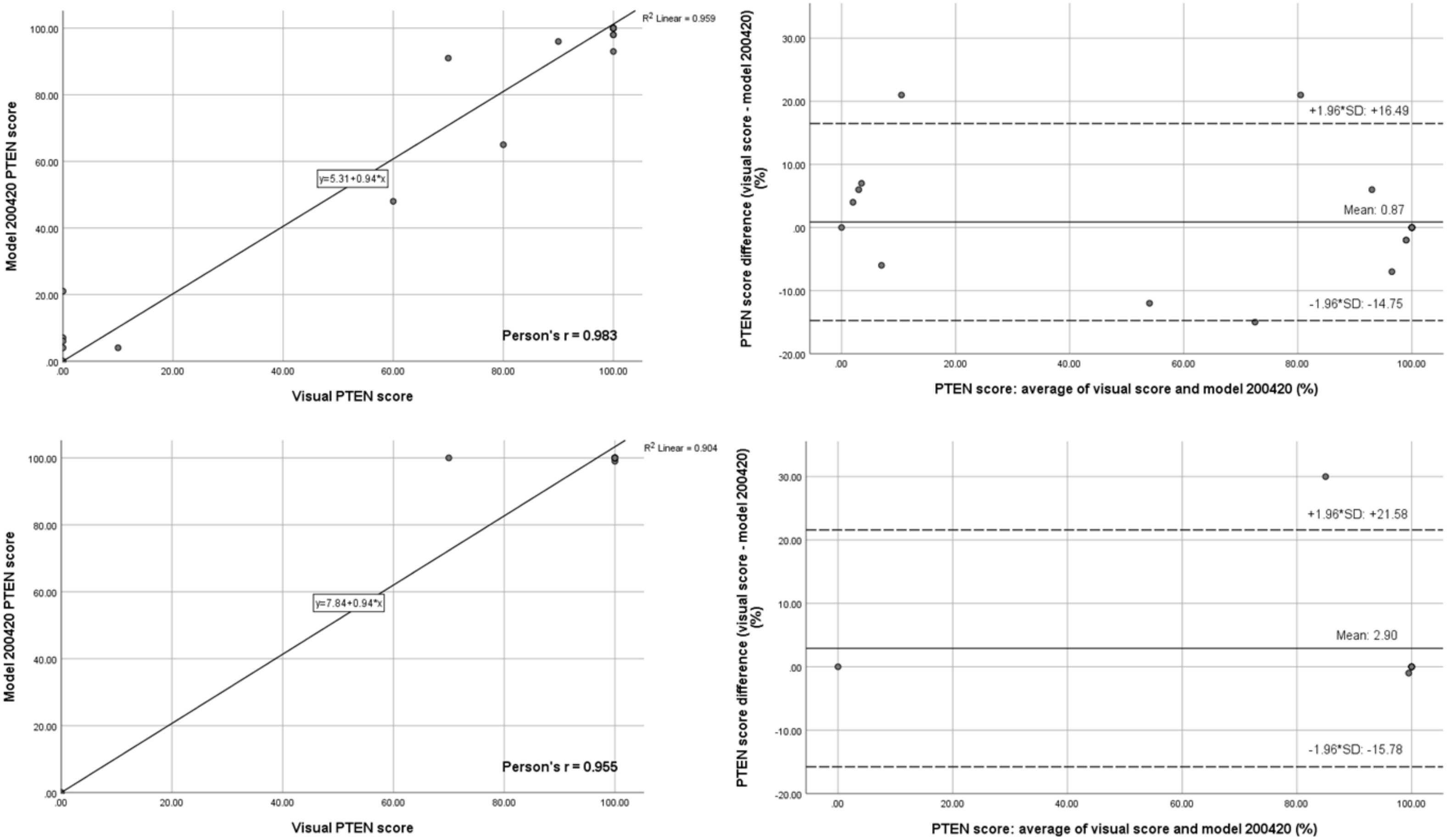

Protocol Fig. 5: Scatterplots with correlation coefficients (left) and Bland-Altman plots of agreement (right) between the model 200420 PTEN scores and the visual PTEN scores in the train subset of 24 WSIs (upper row) and the tune subset of 10 WSIs (lower row). Some of the data points in this figure represent more than one patient due to overlapping PTEN scores.
Abbreviations: PTEN = phosphatase and tensin homologue.

Model 200423

Dataset:

Train subset:

- Number of tiles in data set (including empty tiles): 2160
- Number of empty tiles in data set: 396
- Number of tiles with at least one true PTEN positive tumor nucleus: 987
- Number of tiles with at least one true PTEN negative tumor nucleus: 324

Number of nuclei per class in train subset:

- True PTEN positive tumor nuclei: 51637
- True PTEN negative tumor nuclei: 12217
- False PTEN positive tumor nuclei: 9752
- False PTEN negative tumor nuclei: 920

Tune subset:

- Number of tiles in data set (including empty tiles): 900
- Number of empty tiles in data set: 158
- Number of tiles with at least one true PTEN positive tumor nucleus: 438
- Number of tiles with at least one true PTEN negative tumor nucleus: 57

Number of nuclei per class in tune subset:

- True PTEN positive tumor nuclei: 19420
- True PTEN negative tumor nuclei: 3040
- False PTEN positive tumor nuclei: 5115
- False PTEN negative tumor nuclei: 302

Training:

- 5 classes: background, true PTEN positive tumor nuclei, true PTEN negative tumor nuclei, false PTEN positive tumor nuclei, false PTEN negative tumor nuclei.

- Trained for 40000 iterations.
- Steps: (30000)

Evaluation in the tune subset and the train subset:

Recall: 0.676 (0.878)

Precision: 0.737 (0.849)

mAP: 0.707 (0.824)

Protocol table 2: Confusion matrix of the classification results on the test subset using the 200423 model.

	Predicted true PTEN positive tumor nuclei	*Predicted true PTEN negative tumor nuclei*	*Predicted false PTEN positive tumor nuclei*	*Predicted false PTEN negative tumor nuclei*	*Ground truth object not found or classified as background*	*Number of ground truth objects*
Background	3545	181	1731	23	NA	NA
True positive PTEN tumor nuclei	12674	19	1065	1	5879	19420
True PTEN negative tumor nuclei	7	2611	2	36	396	3040
False PTEN positive tumor nuclei	1512	3	1711	0	1995	5115
False PTEN negative tumor nuclei	4	51	4	26	220	302
Total	17742	2865	4513	86	8490	27877

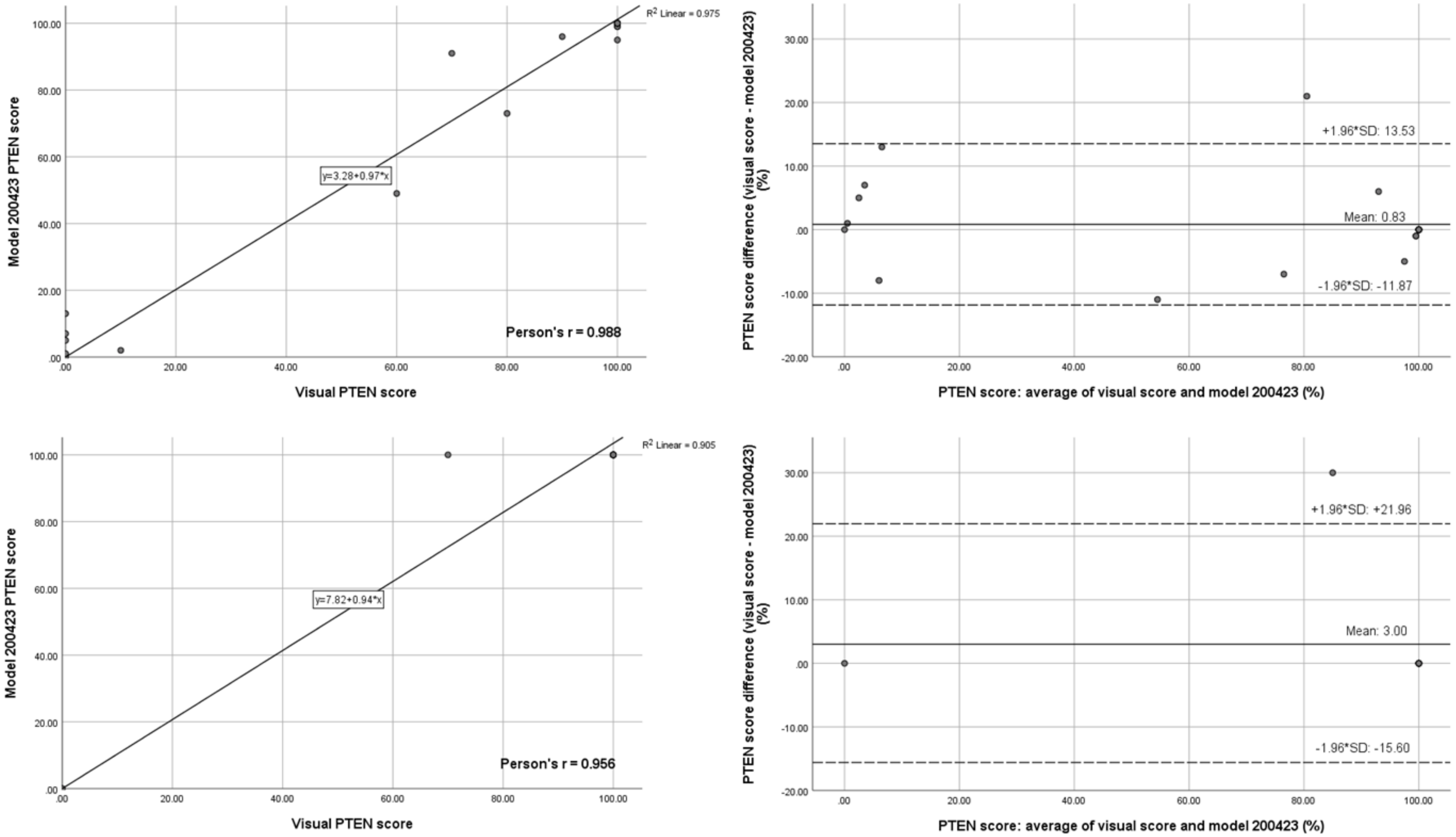

Protocol Fig. 6: Scatterplots with correlation coefficients (left) and Bland-Altman plots of agreement (right) between the model 200423 PTEN scores and the visual PTEN scores in the train subset of 24 WSIs (upper row) and the tune subset of 10 WSIs (lower row). Some of the data points in this figure represent more than one patient due to overlapping PTEN scores.

Abbreviations: PTEN = phosphatase and tensin homologue.

We observed that the 200423 model performed slightly better compared to the 200420 model when comparing the precision (0.737 vs. 0.561) and mAP (0.707 vs. 0.687) in the tune set. Therefore, we decided to use the 200423 model for automatic PTEN scoring in the validation cohort.

4.2 Tumor detector

4.2.1 Dataset, manual annotations and tiling

The WSIs from the 34 patients that were used for training and tuning of the PTEN classifier were also used in the training and tuning of the tumor detector. This subset was expanded by including WSIs from 50 randomly selected patients from the subset of 253 patients (Protocol Fig. 1) to better represent different prostate cancer histologies. Of the 150 WSIs from the 50 patients, a total of 19 WSIs was excluded due: no tumor (n = 5) or technical failure (n = 14) (Protocol Fig. 1). Tumor areas were manually annotated in the remaining 131 WSIs from 49 patients and the 34 WSIs from the 34 patients by the trained expert (KC), based on the annotation of tumor in parallel H&E scans performed by a pathologist (MP) and using a DLine tool (ICGI, OUH, Norway). Large areas with benign glands or non-epithelial tissue were avoided. An additional 20 WSIs from 10 patients that were not included in the subset of 253 patients (Protocol Fig. 1), were included without tumor annotations to allow the network to learn to classify areas with technical failures as non-tumor. PTEN IHC staining for the 20 tissue sections was performed without antibody or with antibody concentration 3x more diluted, in order to make tissue sections with absent or weak PTEN staining (ambiguous staining). The additional patients included in the development of the tumor detector were randomly split on patient level into a training subset containing 129 WSIs (70%) and a test subset containing 56 WSIs (30%) in the same manner as done for the subset of 34 patients (see Section 4.1.1).

The WSIs at full resolution (termed 40x) were split into adjacent, non-overlapping tiles by defining a grid of candidate tiles, each with 800x800 pixels, starting at the upper left corner of the WSI. Excess pixels at the right and bottom of the WSI were ignored, i.e. up to 799 pixels were ignored at right and bottom of the WSI.

The candidate tiles were read into Python as an RGB image using Openslide version 1.1.1 and converted to grayscale images using ITU-R 601-2 luma transform as implemented in the OpenCV library (cvtColor(tile, cv2.COLOR_RGB2GRAY)). If more than 50% of the pixels in a tile had a grayscale value higher than 220, then the tile was considered to be a background tile and excluded from further considerations. The other candidate tiles were classified as either tumor or non-tumor based on the associated manual tumor annotations. If the center position of the tile was inside the tumor annotation, the tile was classified as a tumor tile. To test for this, Shapely polygons were created from the tumor annotation using the Shapely library. As a substitute for the center position of the tile, a Shapely Point with 0-indexed position (400, 400) was used. The Shapely method point.within (polygon) was used to determine if the center position of the tile was inside the tumor annotation or not. If found to be inside the tumor annotation, then the tile was classified as a tumor tile. Otherwise, it was classified as a non-tumor tile. The number of tumor and non-tumor tiles in the train subset and the tune subset were then:

Train subset:

- Total number of candidate non-background tiles: 881418
- Number of tumor tiles: 241170 (27.36%)
- Number of non-tumor tiles: 640248 (72.64%)

Tune subset:

- Total number of candidate non-background tiles: 332211
- Number of tumor tiles: 97587 (29.38%)
- Number of non-tumor tiles: 234624 (70.62%)

4.2.2 Training and tuning of the tumor detector

A classification neural network was trained to classify tiles as tumor or non-tumor using the tumor and non-tumor tile images described in the previous section (Section 4.2.1). Background tiles were not included in training nor tuning, but automatically excluded as they would also be in applications of the tumor detector. As the starting point for our training, we used the Inception v3 model from torchvision.models, which was pre-trained on ImageNet but has the same network architecture as the originally described Inception v3 network [17]. We changed the number of

output classes to 2, representing non-tumor and tumor, and fine-tuned the model using our train subset.

Before a tumor or non-tumor tile in the train subset entered the network, it was read as an RGB image, resized from 800x800 pixels to 299x299 pixels using torchvision.transforms (https://pytorch.org/docs/stable/torchvision/transforms.html) with bilinear interpolation, distorted and normalized. The applied distortion process consisted of precisely the same five distortion steps as used when training the PTEN classifier (see Section 4.1.2). The distorted, resized tile was normalized by subtracting 0.485, 0.456 and 0.406 from the value of the red, green and blue channel, respectively, and dividing the differences by 0.229 0.224 and 0.225 for the red, green and blue channel, respectively. The network was trained on a machine with 4 NVIDIA Titan Xp GPU cards using a batch size of 32 tiles. Tiles were randomly sampled with weights to balance the number of tumor and non-tumor tiles in the mini batches. The network was trained using stochastic gradient descent with cross entropy loss. The base learning rate was 0.001, and the momentum parameter was set to 0.9. Every third epoch, the learning rate was decayed by a factor of 0.1. Because of the weighted random sampling, some tiles were used more than once during an epoch, and some tiles were not used at all during an epoch. The network was trained for 10 epochs. After each epoch, the model was saved and the accuracy (i.e. the ratio between number of correctly classified tiles and the number of classified tiles) was calculated on both the train subset and the tune subset. The model with the highest accuracy on the tune subset was chosen as our final model, which occurred after epoch 2 (Protocol table 3).

Protocol table 3: Accuracy of the model after each epoch of training the tumor detector network.

Epoch	Train subset accuracy	Tune subset accuracy
1	0.911	0.920
2	0.933	0.926
3	0.939	0.924
4	0.946	0.920
5	0.947	0.920
6	0.949	0.922
7	0.949	0.923
8	0.950	0.920
9	0.949	0.920
10	0.950	0.923

A pathologist (MP) visually evaluated heatmaps depicting the predicted tumor areas and observed some WSIs with very small areas spuriously detected as tumor by the automatic tumor detector. We therefore evaluated the effect of excluding predicted tumor areas less than 0.5 mm^2, 1 mm^2 or 4 mm^2 when requiring 8-connectivity within each tumor area. The classification performance on the test subset (Protocol table 4) and visual evaluations of heatmaps indicated that a threshold of 1 mm^2 reduced the number false positive tumor detections without removing too many true positive tumor detections and was therefore included as a part of the tumor detector.

Protocol table 4: Classification performance for automatic tumor detection using different thresholds for excluding very small predicted tumor areas.

Performance metric	No size threshold (train, tune)	Exclude <0.5 mm^2 (train, tune)	Exclude <1 mm^2 (train, tune)	Exclude <4 mm^2 (train, tune)
Accuracy	0.947, 0.926	0.958, 0.937	0.957, 0.938	0.955, 0.933
Sensitivity	0.937, 0.907	0.931, 0.899	0.926, 0.894	0.911, 0.869
Specificity	0.951, 0.934	0.968, 0.953	0.969, 0.956	0.972, 0.959
Positive predictive value	0.878, 0.852	0.916, 0.888	0.919, 0.893	0.924, 0.898
Negative predictive value	0.976, 0.960	0.974, 0.958	0.972, 0.956	0.967, 0.946

The pathologist (MP) visually evaluated the performance of the automatic tumor detector (including the 1 mm^2 threshold to exclude very small predicted tumor areas) in 50 WSIs from the tune subset, by roughly estimating whether the predicted tumor areas are within 20% deviation of the true tumor areas. The automatic tumor detection was found to be satisfactory in all but three WSIs cases. In these three WSIs, the true tumor areas were small, so the percentage of deviation was relatively large although not large in absolute terms (Protocol Fig. 7). All undetected tumor areas were acinar adenocarcinomas.

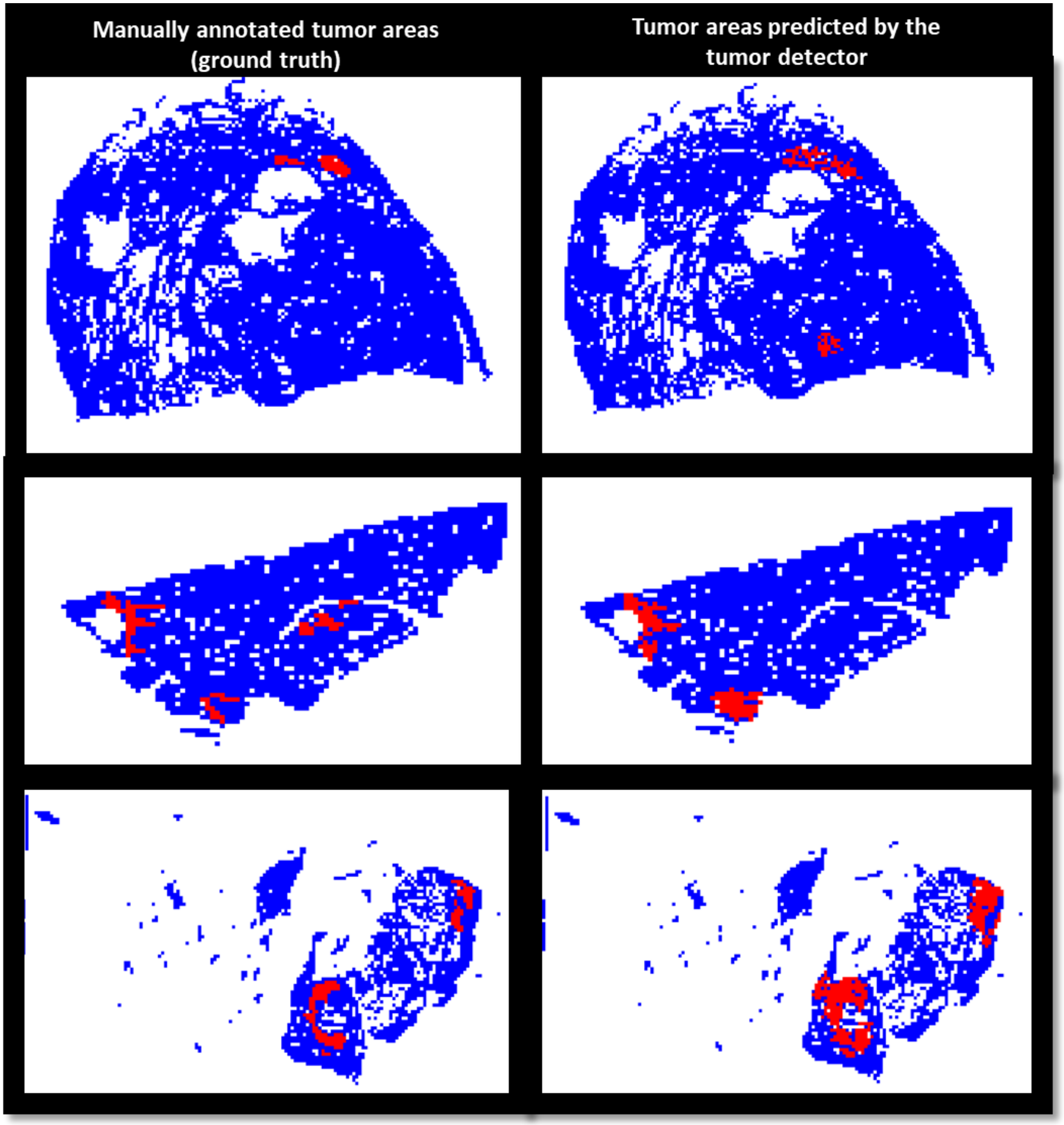

Protocol Fig. 7: Images of the three whole slide images for which tumor areas predicted by the tumor detector (right) were not within 20% deviation of the true tumor areas according to the manual annotations (left).

4.3 Evaluation of the automatic PTEN scoring in the test set

The tumor detector was applied on the test subset of 253 patients (Protocol Fig. 1). Tiles that were classified as tumor tiles were scored automatically by the PTEN classifier. The automatic PTEN scores obtained for each WSI were plotted against the visual scores obtained using scoring protocol 1 and scoring protocol 2 (Protocol Fig. 8). Correlations between visual scoring and automatic scoring were very good (correlation coefficients 0.960 and 0.974). Bland–Altman plots did not reveal any prominent skewness in any data ranges.

For survival analyses, PTEN scores were computed on the patient level as the average of PTEN scores obtained from all evaluated tumor-containing blocks for each particular patient. For comparison with the visual PTEN scores, the resulting automatic PTEN scores on patient level were categorized into 11 groups using intervals of 10%, i.e. 0%, (0%, 10%], (10%, 20%], ..., (80%, 90%] and (90%, 100%]. The categorized PTEN scores were analyzed as continuous variables in Cox proportional hazard models with a categorized PTEN score as the only variable. The hazard ratios (HRs) with 95% confidence intervals (CIs) for a one category increase in the categorized PTEN score obtained with visual and automatic scoring methods are specified in Protocol table 5. It appears that all three PTEN scoring methods provided PTEN scores with similar prognostic value.

Each of the scoring methods provided valid PTEN scores for 249 of the 253 patients in the test subset. The automatic method failed to detect tumor in 12 tissue sections from seven patients. These missed tumor areas were very small, had intermixed benign glands or had minor technical issues (the tissue was fragmented or wrinkled). The automatic method detected tumor in 23 of the 72 tissue sections that were excluded when scored visually. Of these 23 tissue sections, four were excluded because they did not contain tumor, nine because >95% of the tumor area had fallen off or the tumor area was folded, and the remaining 10 because of ambiguous PTEN staining due to very few internal positive controls or weakly stained internal positive controls. Similar HRs and the corresponding 95% CIs for the automatic PTEN scoring method were obtained from the analysis where the scores from these 23 tissue sections were included (HR 1.123, 95% CI 1.059–1.190) compared to the analysis where these scores were excluded (HR 1.122, 95% CI 1.059–1.190). Therefore, we chose to use the automatic PTEN scoring without any manual exclusions in

the independent validation. Also, we considered such an approach as the most objective and convenient in the clinical practice.

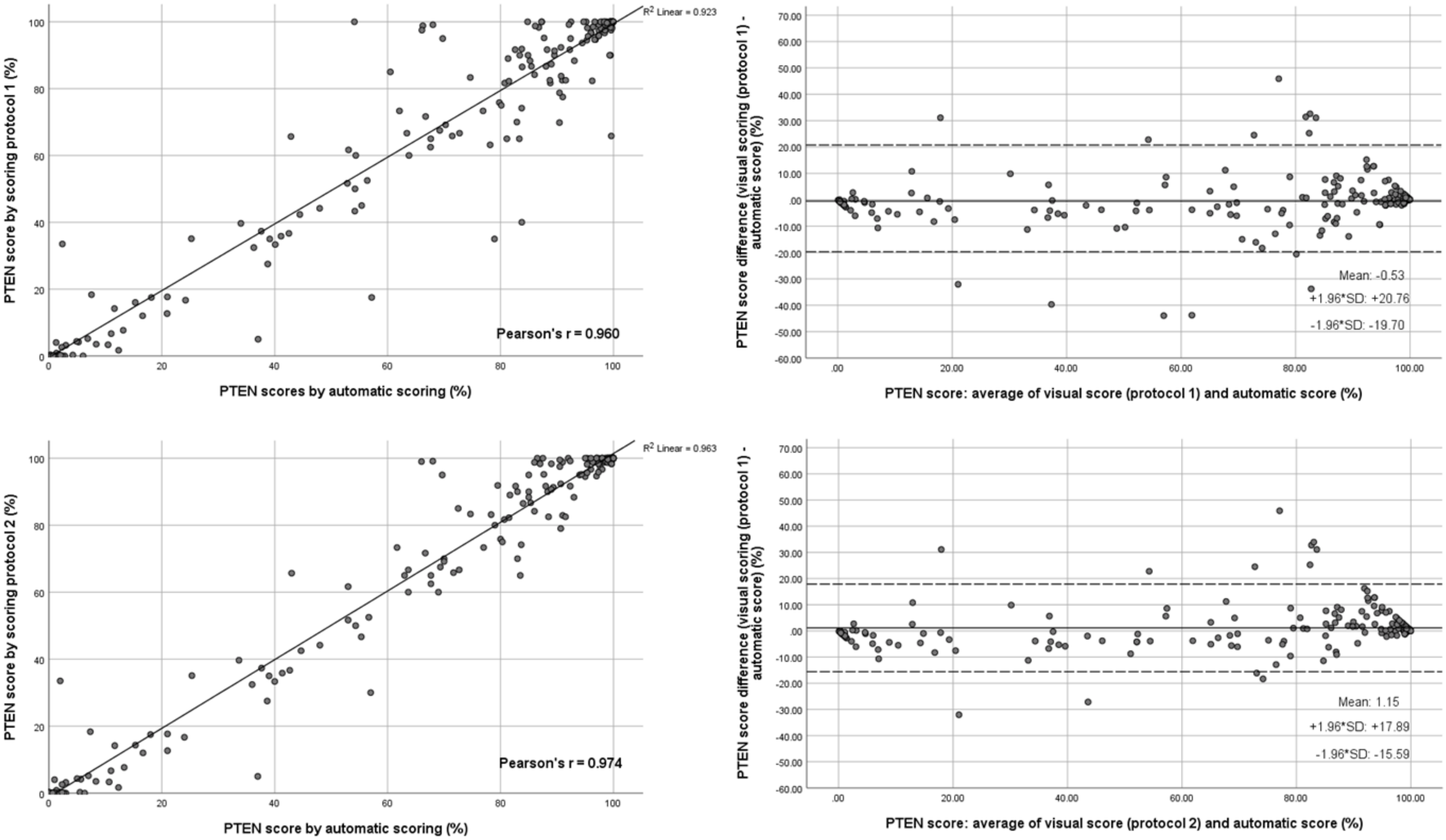

Protocol Fig. 8: Scatterplots with correlation coefficients (left) and Bland-Altman plots of agreement (right) between the automatic PTEN score and the visual PTEN scores obtained by using the scoring protocol 1 (upper row) and between the automatic PTEN score and the visual PTEN scores obtained by using the scoring protocol 2 (lower row). Some of the data points in this figure represent more than one patient due to overlapping PTEN scores.

Abbreviations: PTEN = phosphatase and tensin homologue.

Protocol table 5: Hazard ratios with 95% confidence intervals of the categorized PTEN scores obtained by different scoring methods in univariable analysis of time to recurrence of the 253 patients in the test subset.

PTEN scoring method	HR (95% CI)	P value
Visual scoring using protocol 1	1.128 (1.067–1.193)	<0.001
Visual scoring using protocol 2	1.125 (1.065–1.188)	<0.001
Automatic scoring	1.123 (1.059–1.190)	<0.001
Abbreviations: CI = confidence interval; HR = hazard ratio; PTEN = phosphatase and tensin homologue.		

5. Selection of threshold for dichotomization of PTEN score

Currently, there is no consensus on the threshold for dichotomization of PTEN score. Most of the previous studies have used 90% PTEN positive tumor cells as the threshold [5, 6, 18, 19]. Other studies have used 10% [20–22], 25% [23], 35% [24] or 50% [25] PTEN positive tumor cells as the threshold. In several studies the definition for PTEN positivity or loss was not provided [26–28].

In the test subset of 253 patients, we evaluated different thresholds for dichotomizing the PTEN score in univariable analysis of time to recurrence, time to BCR, time to metastasis and cancer-specific survival (Protocol Fig. 9). The evaluated thresholds were 1%, 2%, 3%, 4%, 5%, 10%, 20%, 30%, 40%, 50%, 60%, 70%, 80%, 90%, 95%, 96%, 97%, 98% and 99%. Based on these results, we decided to use the 50% threshold in the independent validation because it appeared to perform well for all of the endpoints when evaluating the test subset. It should be noted that the test subset comprised mostly patients with advanced disease, and PTEN loss has been observed to be more frequent in tumors with higher stage and GGG [5, 29]. While this might be an argument against using e.g. 10% as the threshold, we consider the 50% threshold to be suited for more contemporary cohorts where patients have less advanced disease at the time of the surgery [21, 24].

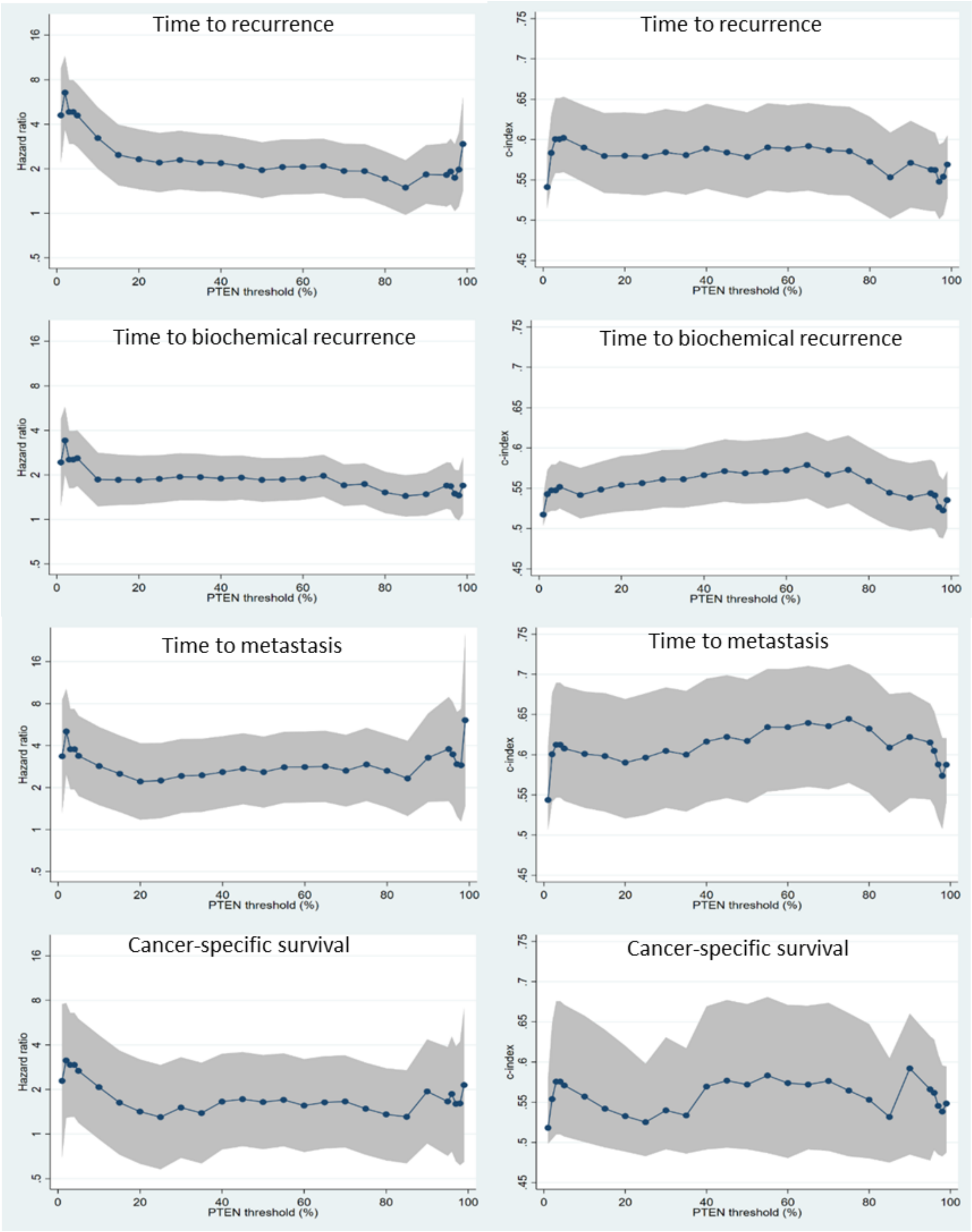

Protocol Fig. 8: Hazard ratios (left) and c-indices (right) of the PTEN score dichotomized by different thresholds when evaluating the test subset of 253 patients. The PTEN score of a patient was calculated by using the automatic method to compute a PTEN score for each of the patient's tumor-containing blocks and then averaging the PTEN scores of the blocks.
Abbreviation: PTEN = phosphatase and tensin homologue.

6. Primary and secondary analyses

1. Primary analysis

The primary analysis aims to evaluate the prognostic value of the deep learning system for automatic quantification of PTEN score on the independent cohort with three tumor-containing blocks for each of the 259 eligible patients, without any manual exclusion of slides or patients. The deep learning system comprise of the automatic tumor detector, which include the 1 mm^2 threshold to exclude very small predicted tumor areas, and a PTEN classifier to predict PTEN positive and PTEN negative tumor nuclei (specifically, the model identified as 200423 will be applied). The PTEN score for each tumor-containing block will be calculated as the ratio between the number of predicted PTEN positive tumor nuclei and the total number of predicted PTEN positive or PTEN negative tumor nuclei. The PTEN score for a patient will be calculated as the average PTEN score of its tumor-containing blocks. Patients with a PTEN score less than 50% will be categorized as PTEN lost, while patients with a PTEN score of at least 50% will be categorized as PTEN present. The prognostic value of this dichotomous biomarker of PTEN status will be analyzed in the primary analysis by computing its hazard ratio (with 95% confidence interval (CI)) in univariable Cox proportional hazard regression analysis with time to BCR, defined as a single PSA ≥0.4 ng/ml, as the endpoint. The selected test for assessing whether PTEN status predicts BCR is the two-tailed Mantel-Cox log-rank test using significance level 0.05. Time to BCR will be calculated from the date of surgery to the first date of BCR or to the date of the last recorded PSA measurement (24th of June 2020).

2. Secondary analyses

The following secondary analyses were predefined before the evaluation in the validation cohort.

1. Evaluate correlation between the automatic and the visual PTEN scores for WSIs using Pearson correlation coefficient with 95% CI and corresponding p value.
2. Categorize the automatic and the visual PTEN scores into 11 groups using intervals of 10%, i.e. 0%, (0%, 10%], (10%, 20%], ..., (80%, 90%] and (90%, 100%]. For each of the scoring methods, compute the HR with 95% CI and corresponding p values of the

categorized PTEN score by analyzing a Cox proportional hazard model with the categorized PTEN score as the only variable (continuous) and using the same endpoint as in the primary analysis.

3. Repeat the primary analysis separately for:
   a) Patients with low risk as given by the CAPRA-S score.
   b) Patients with intermediate risk as given by the CAPRA-S score.
   c) Patients with high risk as given by the CAPRA-S score.
4. Include PTEN status in a multivariable model together with age (continuous), preoperative PSA level (log_2 transformed), GGG (categorical) and the dichotomous pathologic staging parameters EPE, SM, SVI and LNI. Exclude all patients with missing value for any included variable. Compute the HR (with 95% CI) and corresponding p value of PTEN status in analysis of the same endpoint as in the primary analysis.
5. Compute the c-index for PTEN status alone and when integrated with the CAPRA-S score by adding 1 point if PTEN loss in analysis of the same endpoint as in the primary analysis.
6. Compute the c-index for PTEN status alone separately for:
   d) Patients with low risk as given by the CAPRA-S score.
   e) Patients with intermediate risk as given by the CAPRA-S score.
   f) Patients with high risk as given by the CAPRA-S score.
7. Compute HRs with 95% CIs for the combined biomarker of PTEN and DNA ploidy status by analyzing a Cox proportional hazard model with the combined biomarker as the only variable (included as a categorical variable, i.e. the model will consist of the two indicator variables for 1) either non-diploid or PTEN lost but not both and 2) both non-diploid and PTEN lost) and the same endpoint as in the primary analysis. The combined biomarker of PTEN and DNA ploidy status constitutes three risk groups:
   a) diploid and PTEN present,
   b) either non-diploid or PTEN lost, and
   c) non-diploid and PTEN lost.
8. Repeat the secondary analysis numbered 7 separately for:
   a) Patients with low risk as given by CAPRA-S score.
   b) Patients with intermediate risk as given by CAPRA-S score.
   c) Patients with high risk as given by CAPRA-S score.

9. Include the combined biomarker of PTEN and DNA ploidy status (as a categorical variable) in a multivariable model together with age (continuous), preoperative PSA level (log_2 transformed), GGG (categorical) and the dichotomous pathologic staging parameters ECE, SM, SVI and LNI. Exclude all patients with missing value for any included variable. Compute the HRs (with 95% CIs) of the combined biomarker and the P value of the combined biomarker using Wald χ^2 test in analysis of the same endpoint as in the primary analysis.
10. Compute the c-index for the combined PTEN and DNA ploidy status alone and when integrated with the CAPRA-S score by adding 1 point if PTEN loss and 1 point if non-diploid in analysis of the same endpoint as in the primary analysis.
11. Compute the c-index for the combined PTEN and DNA ploidy status separately for:
    a) Patients with low risk as given by the CAPRA-S score.
    b) Patients with intermediate risk as given by the CAPRA-S score.
    c) Patients with high risk as given by the CAPRA-S score.

7. References

1. Cyll K, Ersvær E, Vlatkovic L, et al (2017) Tumour heterogeneity poses a significant challenge to cancer biomarker research. Br J Cancer 117:367–375
2. Epstein JI, Allsbrook WC, Amin MB, Egevad LL (2005) The 2005 International Society of Urological Pathology (ISUP) Consensus Conference on Gleason Grading of Prostatic Carcinoma. Am J Surg Pathol 29:1228–1242
3. Epstein JI (2010) An Update of the Gleason Grading System. J Urol 183:433–440
4. Epstein JI, Egevad L, Amin MB, Delahunt B, Srigley JR, Humphrey PA, Committee G (2016) The 2014 International Society of Urological Pathology (ISUP) Consensus Conference on Gleason Grading of Prostatic Carcinoma: Definition of Grading Patterns and Proposal for a New Grading System. Am J Surg Pathol 40:244–252
5. Lotan TL, Heumann A, Rico SD, Hicks J, Lecksell K, Koop C, Sauter G, Schlomm T (2017) PTEN loss detection in prostate cancer: comparison of PTEN immunohistochemistry and PTEN FISH in a large retrospective prostatectomy cohort. Oncotarget 8:65566–65576
6. Ahearn TU, Pettersson A, Ebot EM, et al (2016) A Prospective Investigation of PTEN Loss and ERG Expression in Lethal Prostate Cancer. J Natl Cancer Inst 108:djv34
7. Bismar TA, Hegazy S, Feng Z, Yu D, Donnelly B, Palanisamy N, Trock BJ (2018) Clinical utility of assessing PTEN and ERG protein expression in prostate cancer patients: a proposed method for risk stratification. J Cancer Res Clin Oncol 144:2117–2125
8. Hedley D, Friedlander M, Taylor I, Rugg C, Musgrove E (1983) Method for analysis of cellular DNA content of paraffin-embedded pathological material using flow cytometry. J Histochem Cytochem 31:1333–1335
9. Cyll K, Callaghan P, Kildal W, Danielsen HE (2015) Preparing for image based DNA ploidy (2015). [Online Video]. 19 Oct Available from:
10. Danielsen HE, Pradhan M, Novelli M (2016) Revisiting tumour aneuploidy - the place of ploidy assessment in the molecular era. Nat Rev Clin Oncol 13:291–304
11. Ersvær E, Hveem TS, Vlatkovic L, et al (2020) Prognostic value of DNA ploidy and automated assessment of stroma fraction in prostate cancer. Int J Cancer 147:1228–1234
12. Punt CJ, Buyse M, Köhne CH, Hohenberger P, Labianca R, Schmoll HJ, Påhlman L, Sobrero A, Douillard JY (2007) Endpoints in adjuvant treatment trials: A systematic review of the literature in colon cancer and proposed definitions for future trials. J Natl Cancer Inst 99:998–1003
13. Cooperberg MR, Hilton JF, Carroll PR (2011) The CAPRA-S score: A straightforward tool for improved prediction of outcomes after radical prostatectomy. Cancer 117:5039–5046
14. Harrell FE, Califf RM, Pryor DB, Lee KL, Rosati RA (1982) Evaluating the yield of medical tests. Jama 247:2543–6

15. Efron B (1987) Better Bootstrap Confidence Intervals. J Am Stat Assoc 82:171–185

16. He K, Gkioxari G, Dollár P, Girshick R (2020) Mask R-CNN. IEEE Trans Pattern Anal Mach Intell 42:386–397

17. Szegedy C, Vanhoucke V, Ioffe S, Shlens J, Wojna Z (2016) Rethinking the Inception Architecture for Computer Vision. Proc IEEE Comput Soc Conf Comput Vis Pattern Recognit 2818–2826

18. Lotan TL, Wei W, Morais CL, et al (2016) PTEN loss as determined by clinical- grade immunohistochemistry assay is associated with worse recurrence-free survival in prostate cancer. Eur Urol Focus 2:180–188

19. Lotan TL, Gumuskaya B, Rahimi H, Hicks JL, Iwata T, Robinson BD, Epstein JI, De Marzo AM (2013) Cytoplasmic PTEN protein loss distinguishes intraductal carcinoma of the prostate from high-grade prostatic intraepithelial neoplasia. Mod Pathol 26:587–603

20. Cuzick J, Yang ZH, Fisher G, et al (2013) Prognostic value of PTEN loss in men with conservatively managed localised prostate cancer. Br J Cancer 108:2582–9

21. Léon P, Cancel-Tassin G, Drouin S, et al (2018) Comparison of cell cycle progression score with two immunohistochemical markers (PTEN and Ki-67) for predicting outcome in prostate cancer after radical prostatectomy. World J Urol 36:1495–1500

22. Mithal P, Allott E, Gerber L, et al (2014) PTEN loss in biopsy tissue predicts poor clinical outcomes in prostate cancer. Int J Urol 21:1209–1214

23. Hamid AA, Gray KP, Huang Y, Bowden M, Pomerantz M, Loda M, Sweeney CJ (2019) Loss of PTEN Expression Detected by Fluorescence Immunohistochemistry Predicts Lethal Prostate Cancer in Men Treated with Prostatectomy. Eur Urol Oncol 2:475–482

24. Jamaspishvili T, Patel PG, Niu Y, et al (2020) Risk stratification of prostate cancer through quantitative assessment of PTEN loss (qPTEN). J Natl Cancer Inst. doi: 10.1093/jnci/djaa032

25. de Bono JS, De Giorgi U, Massard C, et al (2016) PTEN loss as a predictive biomarker for the Akt inhibitor ipatasertib combined with abiraterone acetate in patients with metastatic castration-resistant prostate cancer (mCRPC). Ann Oncol 27:718O-718O

26. Tosoian JJ, Guedes LB, Morais CL, Mamawala M, Ross AE, Marzo AM De, Trock BJ, Han M, Carter HB, Lotan TL (2018) PTEN status assessment in the Johns Hopkins active surveillance cohort. Prostate Cancer Prostatic Dis 22:176–181

27. Leapman MS, Nguyen HG, Cowan JE, Xue L, Stohr B, Simko J, Cooperberg M, Carroll P (2018) Comparing Prognostic Utility of a Single-marker Immunohistochemistry Approach with Commercial Gene Expression Profiling Following Radical Prostatectomy. Eur Urol 74:668–675

28. Lokman U, Erickson AM, Vasarainen H, Rannikko AS, Mirtti T (2018) PTEN Loss but Not ERG Expression in Diagnostic Biopsies Is Associated with Increased Risk of Progression and Adverse Surgical Findings in Men with Prostate Cancer on Active Surveillance. Eur Urol Focus 4:867–873

29. Jamaspishvili T, Berman DM, Ross AE, Scher HI, Marzo AM De, Squire JA, Lotan TL (2018) Clinical implications of PTEN loss in prostate cancer. Nat Rev Urol 15:222–234
