# Supplementary material for: PTEN and DNA Ploidy Status by Machine Learning in Prostate Cancer
Source: Cancers (Basel). 2021 Aug 26;13(17):4291. doi: 10.3390/cancers13174291 (PMC8428363; doi:10.3390/cancers13174291)
Supplement: Supplementary file 1 [file cancers-13-04291-s001.zip › PTEN and DNA ploidy status by machine learning in prostate cancer_supplement.pdf]

Table S1. Clinicopathological characteristics and follow-up for all patients in the test subset and separately for patients with PTEN-high and PTEN-low

Characteristic	All	PTEN-high	PTEN-low	p value*
Patients	253	184	65	
Age at surgery, years	62 (58-67)	62 (57-67)	63 (58-67)	0.79
Recurrence†				0.002
No	167 (66%)	131 (71%)	32 (49%)	
Yes#	86 (34%)	53 (29%)	33 (50%)	
Follow-up time of patients without recurrence†	10.2 (7.2–14.2)	9.9 (7.4-14.1)	12.5 (7.9-14.6)	0.29
Preoperative PSA, ng/ml	13 (7.0-20.0)	12.5 (7.0-20.0)	15.0 (7.0-23.0)	0.14
Missing	2 (1%)	2 (1%)	0	
Preoperative PSA				0.19
≤6 ng/ml	57 (23%)	41 (22%)	15 (23%)	
>6 ng/ml and ≤10 ng/ml	48 (19%)	41 (22%)	7 (11%)	
>10 ng/ml and ≤20 ng/ml	84 (33%)	57 (31%)	24 (37%)	
>20 ng/ml	62 (25%)	43 (24%)	19 (29%)	
Missing	2 (1%)	2 (1%)	0	
Gleason grade group				0.001
1 (GS 6)	11 (4%)	9 (5%)	0	
2 (GS 3+4)	92 (36%)	77 (42%)	13 (20%)	
3 (GS 4+3)	77 (30%)	51 (28%)	26 (40%)	
4 (GS 8)	44 (17%)	25 (13%)	19 (29%)	
5 (GS 9-10)	29 (11%)	22 (12%)	7 (11%)	
Extraprostatic extension				<0.0001
Absent	55 (22%)	49 (26%)	3 (5%)	
Present	196 (78%)	133 (73%)	62 (95%)	
Missing	2 (1%)	2 (1%)	0	
Surgical margin				0.23
Negative	91 (36%)	70 (38%)	19 (29%)	
Positive	162 (64%)	114 (62%)	46 (71%)	
Seminal vesicle invasion				<0.0001
Absent	187 (74%)	148 (80%)	35 (54%)	
Present	66 (26%)	36 (20%)	30 (46%)	
Lymph node involvement				0.37
Absent	239 (94%)	175 (95%)	60 (92%)	
Present	14 (6%)	9 (5%)	5 (8%)	
CAPRA-S risk group				0.008
Low	30 (12%)	25 (14%)	3 (5%)	
Intermediate	87 (35%)	69 (38%)	17 (26%)	
High	132 (53%)	86 (47%)	45 (69%)	
Missing	4 (2%)	4 (2%)	0	

Data are median (IQR) or n (%). CAPRA-S = Cancer of the Prostate Risk Assessment Postsurgical; GS = Gleason score; IQR = interquartile range; PSA = prostate-specific antigen; PTEN = phosphatase and tensin homolog. *Fisher's exact (categorical variables) or Mann-Whitney *U* (continuous variables) test evaluated using only non-missing values. † Defined as locoregional recurrence (confirmed by histological biopsies or ultrasound), distant metastasis (detected by skeletal scintigraphy) or death from prostate cancer (based on death certificate). #Of the 86 patients, 36 (42%) patients had local recurrence without metastatic disease, 22 (26%) patients developed metastatic disease, 23 (27%) patients had both local recurrence and metastatic disease and five (6%) patients died of prostate cancer without having a previous record of recurrence.

Table S2. Uni- and multivariable analyses of time to biochemical recurrence including centrally reviewed Gleason scores.

Variable	Group	Univariable analysis		Multivariable analysis*	
		HR (95% CI)	p value	HR (95% CI)	p value
**A) Standard clinicopathologic parameters**					
Ploidy and PTEN status			<0.0001		0.10
	Diploid and PTEN-high	ref.		ref.	
	Non-diploid or PTEN-low	1.94 (1.15-3.30)		1.12 (0.61-2.04)	
	Non-diploid and PTEN-low	4.63 (2.50-8.57)		2.22 (1.04-4.74)	
Age at surgery	10-year increment	1.51 (1.00-2.26)	0.048	0.82 (0.52-1.29)	0.39
Preoperative PSA	$\log_2$(1+ng/ml) increment	2.39 (1.74-3.28)	<0.0001	2.57 (1.63-4.06)	<0.0001
Gleason grade group			<0.0001		0.0001
	1 (GS 6)	0.00 (0.00-∞)		0.00 (0.00-∞)	
	2 (GS 3+4)	ref.		ref.	
	3 (GS 4+3)	1.66 (0.85-3.22)		1.25 (0.61-2.55)	
	4 (GS 8)	1.70 (0.51-5.67)		2.59 (0.76-8.87)	
	5 (GS 9-10)	10.94 (6.31-18.95)		4.76 (2.37-9.54)	
Extracapsular extension	Present vs. Absent	3.97 (2.45-6.44)	<0.0001	1.57 (0.87-2.82)	0.14
Surgical margins	Positive vs. Negative	2.90 (1.80-4.67)	<0.0001	2.44 (1.41-4.21)	0.0014
Seminal vesicle invasion	Present vs. Absent	5.39 (3.22-9.02)	<0.0001	1.42 (0.76-2.64)	0.27
Lymph node involvement	Present vs. Absent	6.25 (2.67-14.60)	<0.0001	1.35 (0.51-3.56)	0.55
**B) CAPRA-S risk groups**					
Ploidy and PTEN status			<0.0001		0.017
	Diploid and PTEN-high	ref.		ref.	
	Non-diploid or PTEN-low	1.94 (1.15-3.30)		1.20 (0.69-2.09)	
	Non-diploid and PTEN-low	4.63 (2.50-8.57)		2.53 (1.32-4.82)	
CAPRA-S risk group					
	Low (score 0–2)	ref	<0.0001	ref.	<0.0001
	Intermediate (score 3–5)	4.17 (1.45-12.03)		4.09 (1.42-11.80)	
	High (score ≥ 6)	22.06 (7.88-61.76)		18.71 (6.57-53.29)	

CAPRA-S = Cancer of the Prostate Risk Assessment Postsurgical; CI = confidence interval; GS = Gleason score; HR = hazard ratio; PSA = prostate-specific antigen; PTEN = phosphatase and tensin homolog. *Of the 259 patients included in univariable analyses (71 with event and 188 without event), 251 (69 with event and 182 without event) had complete data and were included in the multivariable analysis.

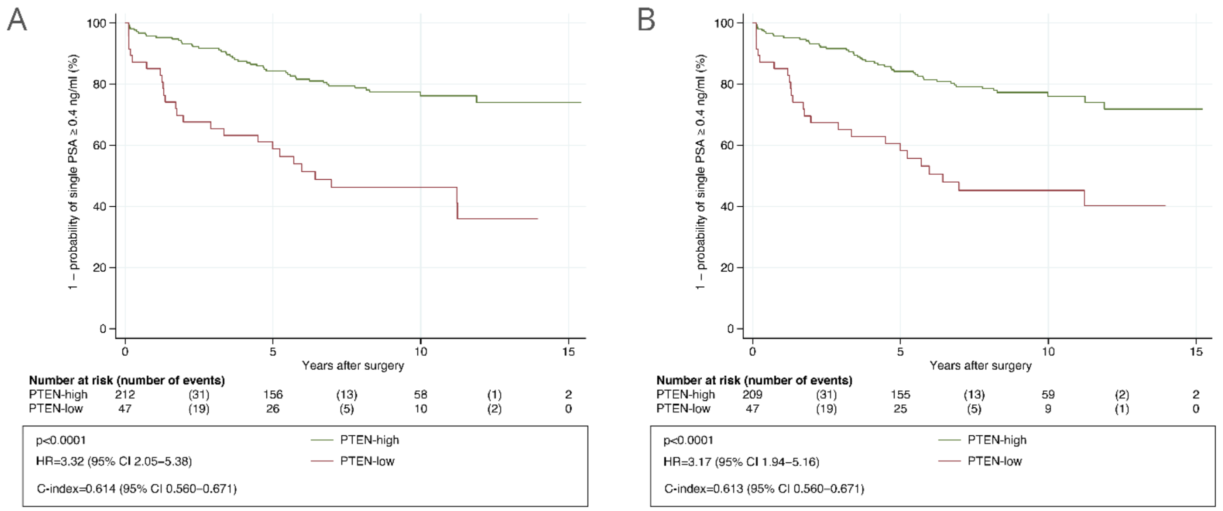

Figure S1. Kaplan-Meier analysis of time to biochemical recurrence after radical prostatectomy in the validation cohort stratified by PTEN status determined using average PTEN scores for each patient, scored by **(A)** the first observer (K.C.) and **(B)** the second observer (E.E.).

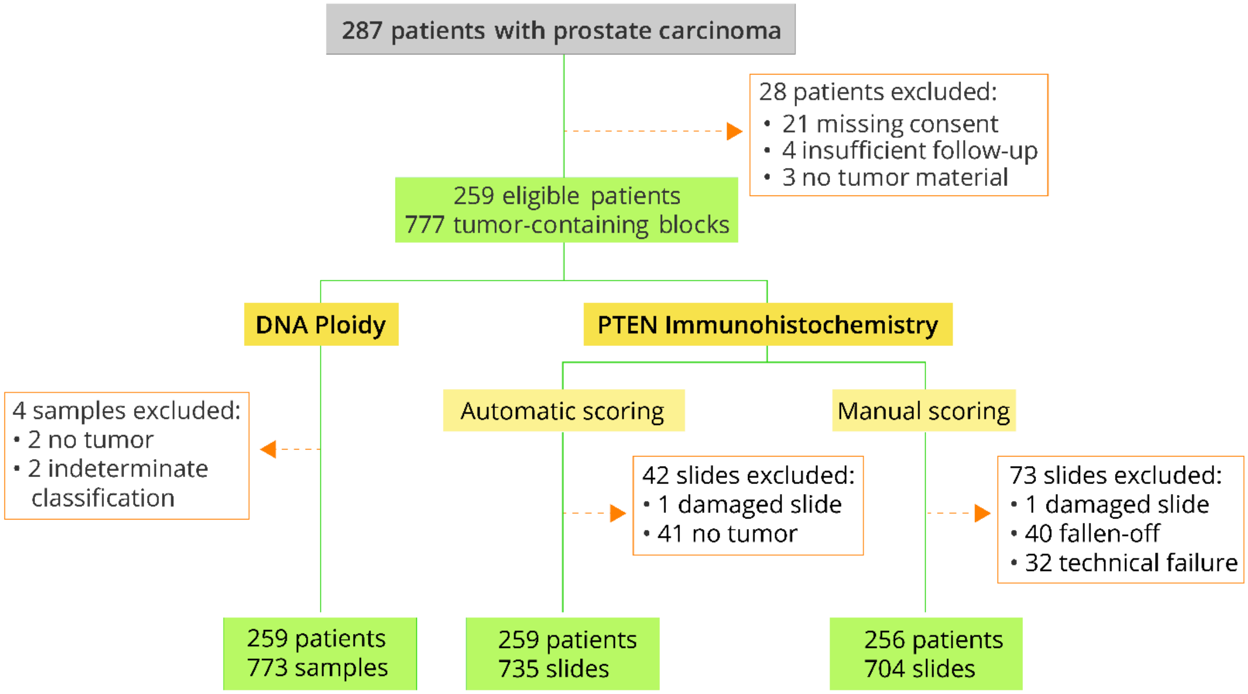

Figure S2. Overview of analyzed patient material and applied methods in the validation cohort. PTEN immunohistochemistry and DNA ploidy were performed using three separate tumor-containing tissue blocks for each patient. Following DNA ploidy analysis, four samples were excluded; two samples had insufficient tumor material for monolayer preparation, whilst two could not be classified due to having less than 200 nuclei. Of the 777 PTEN-immunohistochemically stained slides, one was damaged and thus whole slide images (WSIs) were obtained from 776 slides. When scoring manually, 40 WSIs were excluded because ≥95% of the tumor area had fallen off during sample preparation and 32 excluded due to ambiguous staining. The automatic scoring method did not detect tumor in 41 WSIs.

Abbreviations: PTEN = phosphatase and tensin homologue.

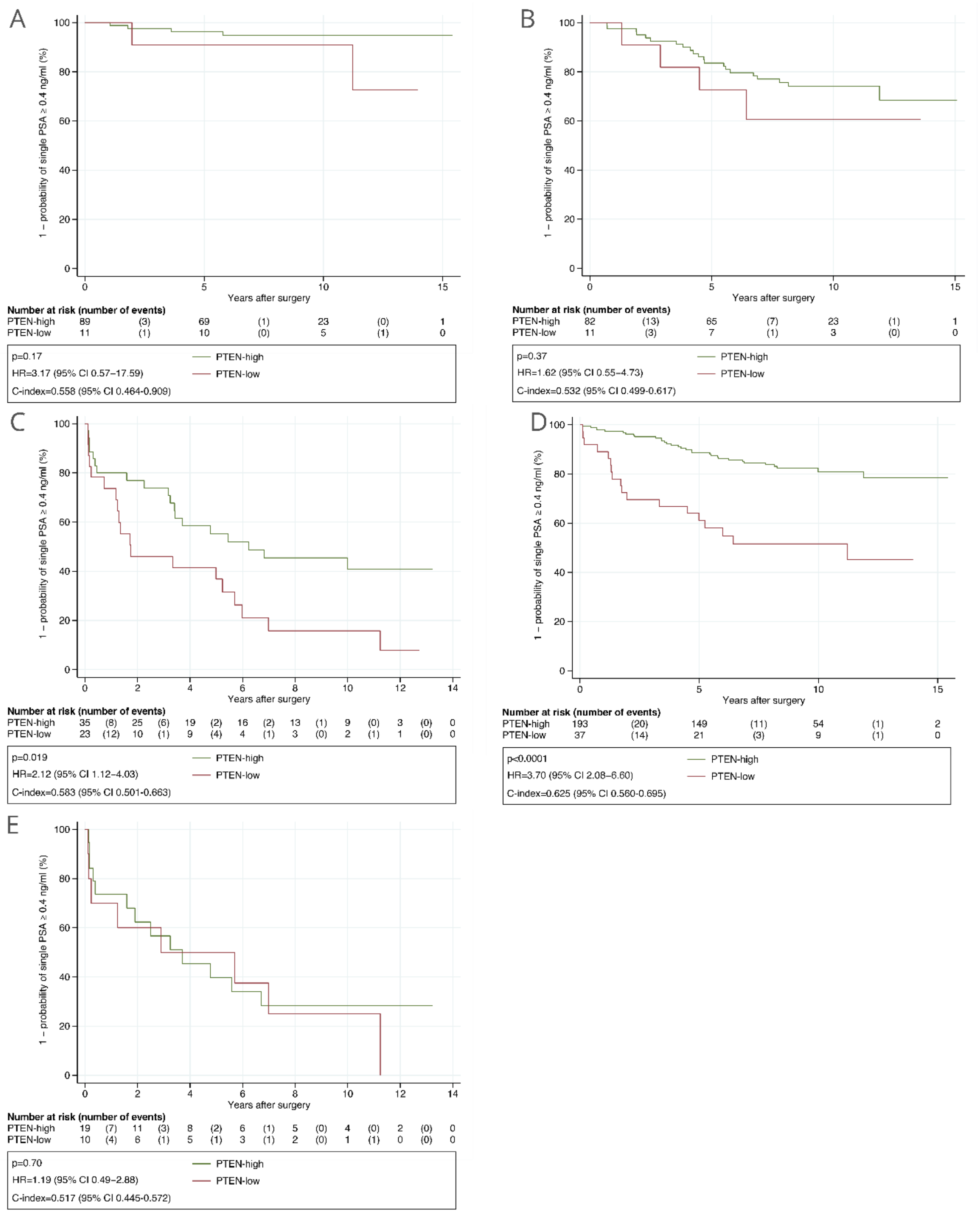

Figure S3. Kaplan-Meier analysis of time to biochemical recurrence after radical prostatectomy stratified by the automatically assessed PTEN status in the validation cohort. **(A)** Patients with low risk as given by CAPRA-S score. **(B)** Patients with intermediate risk as given by CAPRA-S score. **(C)** Patients with high risk as given by CAPRA-S score. **(D)** Patients with GGG ≤3 tumors. **(E)** Patients with GGG >3 tumors. Routine Gleason scores were used in the analyses.

Abbreviations: CAPRA-S = Cancer of the Prostate Risk Assessment Post-Surgical score; C-index= concordance index; CI = confidence interval; GGG = Gleason grade group; HR = hazard ratio; PTEN = phosphatase and tensin homolog.

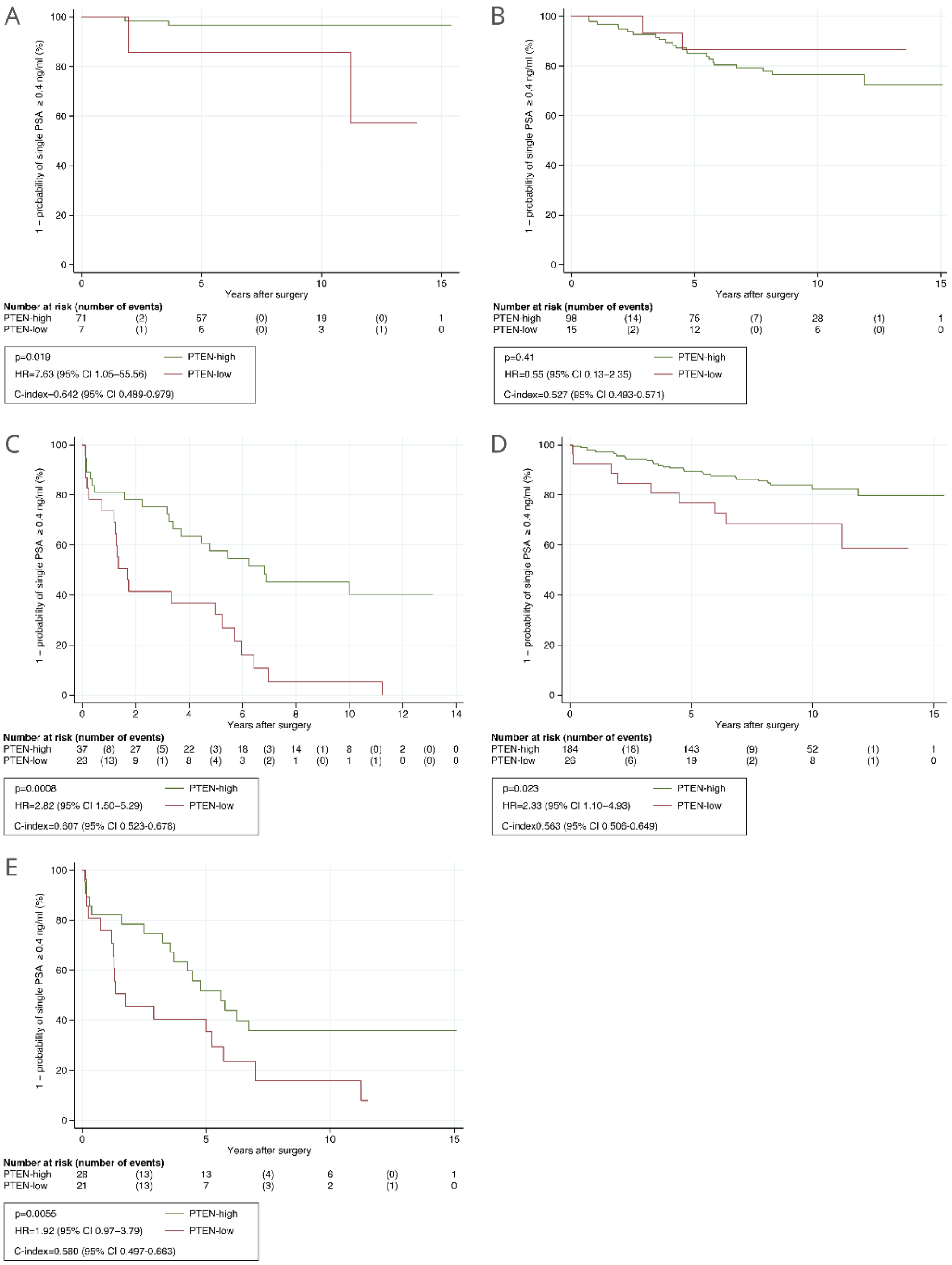

Figure S4. Kaplan-Meier analysis of time to biochemical recurrence after radical prostatectomy stratified by the automatically assessed PTEN status in the validation cohort. **(A)** Patients with low risk as given by CAPRA-S score. **(B)** Patients with intermediate risk as given by CAPRA-S score. **(C)** Patients with high risk as given by CAPRA-S score. **(D)** Patients with GGG ≤3 tumors. **(E)** Patients with GGG >3 tumors. Centrally reviewed Gleason scores were used in the analyses.

Abbreviations: CAPRA-S = Cancer of the Prostate Risk Assessment Post-Surgical score; C-index= concordance index; CI = confidence interval; GGG = Gleason grade group; HR = hazard ratio; PTEN = phosphatase and tensin homolog.

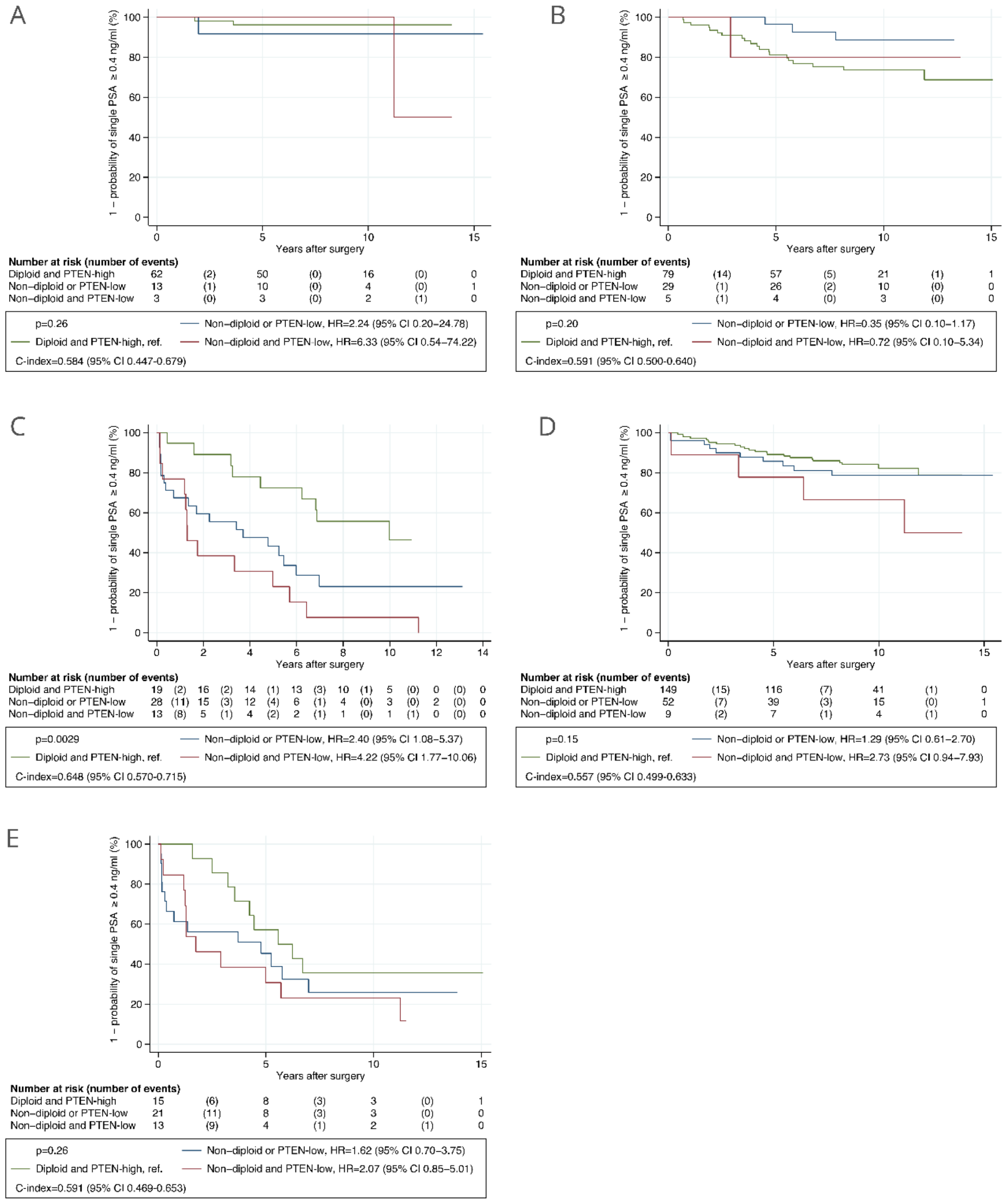

Figure S5. Kaplan-Meier analysis of time to biochemical recurrence after radical prostatectomy stratified by the combined automatically assessed PTEN and DNA ploidy status in the validation cohort. **(A)** Patients with low risk as given by CAPRA-S score. **(B)** Patients with intermediate risk as given by CAPRA-S score. **(C)** Patients with high risk as given by CAPRA-S score. **(D)** Patients with GGG ≤3 tumors. **(E)** Patients with GGG >3 tumors. Centrally reviewed Gleason scores were used in the analyses.
Abbreviations: CAPRA-S = Cancer of the Prostate Risk Assessment Post-Surgical score; C-index= concordance index; CI = confidence interval; GGG = Gleason grade group; HR = hazard ratio; PTEN = phosphatase and tensin homolog.
